# Unmet needs for non-communicable diseases and sexual and reproductive health services among women of reproductive age in low-and-middle-income countries: evidence from the Demographic and Health Surveys

**DOI:** 10.1136/bmjopen-2025-105422

**Published:** 2026-02-09

**Authors:** Yishu Yin, Yeting Du, Zhi-Jie Zheng, Minghui Ren, Minmin Wang, Yinzi Jin

**Affiliations:** 1 School of Public Health, Peking University, Beijing, China; 2 School of Health Humanities, Peking University, Beijing, China; 3 Department of Global Health, School of Public Healt, Peking University, Beijing, China; 4 Institute for Global Health, Peking University, Beijing, China; 5 China Center for Health Development Studies, Peking University, Beijing, China

**Keywords:** Health, Postpartum Women, Chronic Disease, Reproductive medicine, Health Services Accessibility

## Abstract

**Abstract:**

**Introduction:**

Despite international efforts to address women’s long-term health and well-being, significant gaps in sexual and reproductive health (SRH) services and non-communicable diseases (NCDs) prevention remain, particularly in low-and-middle-income countries (LMICs).

**Methods:**

We analysed data from 726 278 women aged 15–49 from six national surveys (2017–2021, Benin, Cameroon, Gabon, India, Madagascar and Mauritania) on unmet needs for NCD prevention (blood pressure, glucose, cervical cancer screening) and SRH services (contraception, antenatal, postnatal care). Unmet needs prevalence was calculated as the percentage of participants with specific unmet needs and estimated across demographics and socioeconomic groups using multivariable logistic regression models.

**Results:**

Unmet needs were strikingly high for NCD prevention: 36.6% for blood pressure, 70.0% for blood glucose and 98.5% for cervical cancer screening. In contrast, unmet needs for contraception, antenatal care and postnatal care were relatively lower: 7.5%, 14.5% and 14.5%, respectively. Significant variations were observed across countries. India had the lowest unmet needs for SRH services: 6.7% for contraception, 13.1% for antenatal care and 13.1% for postnatal care. Gabon had lower unmet needs for prenatal (16.8%) and postnatal care (14.8%) compared with other African countries and the lowest unmet need for cervical screening at 84.7% (95% confidential interval 83.1% to 86.2%), over 10 percentage points lower than others. Furthermore, socioeconomic factors like higher education, better economic status, healthcare access, insurance and internet use significantly lowered unmet needs, especially for antenatal and postnatal care. Employed women had higher unmet needs for antenatal (35.7%) and postnatal (37.3%) care than unemployed women (28.1%, 27.8%) but lower for NCDs prevention (98.9%, 71.8%) under two definitions than unemployed women (99.3%, 79.2%).

**Conclusion:**

This study highlights the urgent need to address high unmet needs for NCD prevention among women in LMICs, particularly cervical cancer screening. Unmet SRH needs are also a major concern, given significant disparities across countries. Especially, governments should prioritise measures to focus on vulnerable groups.

**STRENGTHS AND LIMITATIONS OF THIS STUDY**

- This study provides a novel comparative analysis of unmet needs for both non-communicable diseases (NCDs) prevention and sexual and reproductive health (SRH) services among a large, multinational sample of 726 278 women in low-and-middle-income countries (LMICs), addressing a critical knowledge gap.
- A major strength is that the study employed two alternative definitions of unmet NCD prevention needs, thereby strengthening the robustness of the analysis.
- This study examines how socioeconomic determinants differentially influence unmet needs for SRH versus NCD prevention services.
- A primary limitation is the reliance on secondary data, which precluded the investigation of the specific reasons (eg, financial, logistical, cultural) underlying the observed profound unmet needs.
- The generalisability of the findings may be constrained as the analysis was confined to six LMICs with available data.

## Introduction

 The long-term health and well-being of women have been recognised as a global commitment in sustainable development, with international efforts to address both sexual and reproductive health (SRH) needs and the long-term healthcare needs for non-communicable diseases (NCDs). World Health Day 2025, with the theme *Healthy beginnings, hopeful futures*, calls on governments and the health community to intensify their efforts to eliminate preventable maternal and newborn deaths, and to prioritise women’s longer-term health and well-being. The significance of SRH in global health agendas has been well-acknowledged.^1^ Notwithstanding the progress that has been made, SRH remains a pressing health issue, as evidenced by the stagnation in the global maternal mortality. In 2020 alone, an estimated 287 000 women died from causes related to pregnancy, childbirth and the postpartum period, with the majority of these deaths occurring among women from socioeconomically disadvantaged backgrounds.^2 3^ NCDs, including cardiovascular diseases, cancers, chronic respiratory diseases and diabetes, have emerged as significant challenges to women’s well-being.^4^ Cardiovascular diseases and cancer are the leading causes of mortality among women on a global scale.^5^,^7^ Among these, breast cancer is a particularly salient example of a prevalent cancer. By the end of 2020, the number of women living with a breast cancer diagnosis within the previous 5 years had reached 7.8 million, while 685 000 deaths from the disease occurred in the same period.^8^ Another salient issue is gestational diabetes, the most prevalent pregnancy complication worldwide, affecting one in seven pregnant women.^9^ Women diagnosed with gestational diabetes face a 10-fold higher risk of developing type 2 diabetes later in life, and they are more likely to experience hypertension, dyslipidaemia, obesity and fatty liver disease.^10^

Despite the global focus on improving women’s health, significant gaps remain in healthcare access and delivery, especially in low-and-middle-income countries (LMICs). In settings with limited resources, the procurement and utilisation of effective contraception and reproductive health services are encumbered by numerous obstacles. For instance, while approximately 99% of pregnant women in high-income and upper-middle-income countries receive assistance from skilled midwives, doctors or nurses during childbirth, the proportions in LMICs are only 68% and 78%, respectively.^11^ In regard to the prevention and management of NCDs, the WHO has issued several recommendations, including that all adults regularly check their blood pressure to detect and manage hypertension in a timely manner,^12^ that high-risk groups (including those who are obese, have high blood pressure, high cholesterol or are people who smoke) regularly measure their blood glucose levels to assess treatment effectiveness and reduce the occurrence of complications,^13^ and that the general population undergoes cervical cancer screening every 5–10 years starting at the age of 30.^14^ Nevertheless, the availability and accessibility of these healthcare services were limited in LMICs are constrained, thereby hindering global progress in the prevention and management of NCDs.

The concept of ‘unmet needs’ in healthcare refers to the discrepancy between the health services required by a population and those actually received,^15^ manifesting across four critical stages: screening, diagnosis, treatment and control.^16^ Addressing unmet needs is imperative for achieving universal health coverage, a pivotal component of the sustainable development goals and a fundamental commitment by governments worldwide to ensure all residents have access to high-quality health services.^17 18^ In the context of women’s health, unmet needs manifest in various forms, including but not limited to: lack of access to contraception, inadequate antenatal and postnatal care and insufficient management of NCDs.^19^ These unmet needs are indicative of systemic gaps and inequities in healthcare coverage, adversely impacting women’s health outcomes. Addressing these unmet needs in women’s health is crucial for better achieving the goals of universal health coverage. Nevertheless, significant obstacles persist in addressing these unmet needs, particularly due to the absence of measurement and comparative analysis concerning unmet needs associated with the long-term health and well-being of women of reproductive age. First, women’s health research has historically concentrated on SRH, frequently eclipsing the pressing need for NCD prevention.^5 20^ Second, while extant studies have examined the socioeconomic factors influencing women’s access to NCD services and SRH services, a comparative analysis between the two is lacking.^21 22^

To address these research gaps, the present study aimed to investigate whether the proportion of unmet needs for NCD prevention is higher than that for contraception, antenatal care and postnatal care among 726 278 women of reproductive age (15–49 years) in six LMICs: Benin, Cameroon, Gabon, India, Madagascar and Mauritania. The analysis of data from nationally representative surveys was undertaken to compare the prevalence of unmet needs across various health domains and to identify the demographic and socioeconomic factors influencing access to healthcare. This approach was adopted to pinpoint vulnerable population groups among women of reproductive age. The findings of this study will provide key insights into the health needs of women in LMICs and inform the development of targeted interventions to reduce health disparities and improve health outcomes.

## Methods

### Data sources

Data were obtained from the Demographic and Health Surveys (DHS),^23^ which is a large, global household survey collecting detailed data on population, health and nutrition. It provides an important statistical resource for the analysis of key indicators such as fertility, reproductive health, child health and supports comparative studies across countries and time.^24^ Considering that DHS began to collect information on NCD services in 2015,^25^ we adopted the following inclusion criteria: (1) conducted after 2015 and data set was accessible through the DHS database, (2) respond to three NCD services: ‘ever had blood pressure measured’, ‘ever had blood sugar measured’ and ‘tested for cervical cancer’, (3) respond to questions like ‘unmet need for contraception’, (4) respond to questions of antenatal care including ‘prenatal: no one’, ‘timing of first antenatal check (months)’, ‘number of antenatal visits during pregnancy’ and ‘place of delivery’ and (5) consisted of questions and valid responses for postnatal care ‘baby postnatal check within 2 months’.

Six countries had data meeting the inclusion criteria and were included in our study: Benin (2017), Cameroon (2018), Gabon (2019), India (2019), Madagascar (2021) and Mauritania (2019). Participants with missing data on key variables (blood pressure measuring, blood glucose measuring, cervical cancer screening, contraceptive use, antenatal care and postnatal care) were excluded from the analysis. Detailed information about the number of excluded participants and reasons for exclusion is provided in online supplemental S1 figure.

### Definitions

To assess the extent of unmet needs in various health domains, we employed the unmet need level as a key indicator.


$$Unmet\;need\;level=\frac{Number\;of\;participants\;with\;specific\;unmet\;need\left(s\right)}{Total\;number\;of\;participants}\times 100\%$$

The analysis of unmet needs level in first requires the identification of individuals with specific unmet needs. Our definition of unmet needs primarily references the essay by Allin *et al*, defining it as the need for appropriate healthcare services that individuals have not received. Such needs may remain unmet for various reasons, including: unperceived unmet need, subjective, chosen unmet need, subjective, not-chosen unmet need, subjective, clinician-validated unmet need and subjective unmet expectations.^26^

*Unmet needs in NCD prevention:* Following Alattas’s study,^27^ our definition of unmet needs in NCD prevention is based on whether individuals have received the following screening services: blood pressure measuring, blood glucose measuring or cervical cancer screening. Besides, though the recommended age for cervical cancer screening is 25–64, the DHS only collected individual data for women aged 15–49. Consistent with other DHS-based studies,^21 28 29^ our study focused on the included groups. For lack of a standardised definition of NCDs prevention and to ensure the robustness of our analysis, we adopted two definitions of unmet needs in NCDs prevention: (i) defined as individuals who have not received at least one of the above screening services; and (ii) defined as individuals who have not received two or more of the above screening services. These definitions allow us to capture the extent of unmet needs comprehensively while accounting for varying levels of service utilisation.

*Unmet contraceptive needs:* Participants who reported ‘unmet need for spacing, unmet need for limiting’ are classified as having unmet contraceptive needs, while the met need for contraception was defined as when a woman reported ‘using for spacing’ and ‘using for limiting’. This definition followed the previous consensus to measure the unmet needs.^2630^,^33^

*Unmet antenatal care needs:* We define it as pregnant women who receive fewer than four antenatal check-ups, have their first check-up after the third month of pregnancy or give birth at home or in a non-public facility, unless they have received adequate antenatal care. This definition referenced the WHO recommendations^34 35^ and included the place of delivery to more comprehensively assess unmet needs in antenatal care.

*Unmet postnatal care needs:* In this study, individuals who do not receive a postnatal check-up within 2 months after childbirth were defined as having unmet postnatal care needs. This definition is based on the WHO’s recommendation that the first 6 weeks postpartum are a critical period for ensuring the survival of newborns and mothers.^36^

The details of the questions used to determine participants’ needs for each variable are provided in online supplemental S1 table.

### Statistical analysis

The level and 95% confidential interval (CI) of unmet needs were estimated across various demographic and socioeconomic strata. These strata included age groups (15–19, 20–24, 25–29, 30–34, 35–39, 40–44 and 45–49 years), education level (no education, primary, secondary and higher), employment status (currently working vs not working), marital status (never in union, currently in union/living with a partner, formerly in union/living with a partner), wealth index for urban and rural areas (poorest, poorer, middle, richer and richest), perceived ease of obtaining medical help for oneself (no problem, big problem and not a big problem), health insurance coverage (yes vs no) and internet usage (never vs yes).

The prevalence of unmet needs for NCD prevention, contraception, antenatal care and postnatal care, along with their 95% CIs, was also calculated. To compare the prevalence of unmet needs across different health domains, one-sided *F*-tests were applied for categorical variables. Multivariable logistic regression models were employed to assess the association between unmet needs and various sociodemographic factors (age, education level, respondent currently working status, current marital status, wealth index, getting medical help for self, whether covered by health insurance, use of internet). All statistical analyses were performed using Stata V.18.0 (StataCorp LLC, College Station, Texas, USA). A p value <0.05 was considered statistically significant.

### Ethical considerations

The data analysed in this study were accessed from the freely available open-source DHS data sets. Permission for use was granted to the principal investigator and all collaborators, thus no ethical application was required.

### Patient and public involvement

Not applicable.

## Results

### Sample characteristics

The study included a total of 726 278 women of reproductive age (15–49 years) from six LMICs: Benin, Cameroon, Gabon, India, Madagascar and Mauritania. The distribution of participants across age groups, education levels, wealth quintiles and other demographic and socioeconomic characteristics is summarised in table 1. The majority of the sample (93.7%) was from India, with smaller proportions from Madagascar (1.3%), Cameroon (1.8%), Benin (1.0%), Gabon (0.9%) and Mauritania (1.1%). In terms of age distribution, 17.1% of women were aged 15–19 years, 16.5% were 20–24 years and 16.3% were 25–29 years, with smaller proportions in older age groups. Regarding education, 49.6% of women had completed secondary education, while 22.7% had no formal education. Approximately 71.6% of women were currently married or living with a partner, and 70.9% lacked health insurance coverage.

**Table 1:** Distribution of the study population by country

Variables	Total	Benin	Cameroon	Gabon	India	Madagascar	Mauritania
Sample size	726 277	7706	13 616	6507	680 893	9597	7959
Age in 5-year groups							
15–19	124 658 (17.2)	1617 (21.0)	3309 (24.3)	1127 (17.3)	114 575 (16.8)	2106 (22.0)	1922 (24.2)
20–24	120 470 (16.6)	1423 (18.5)	2417 (17.8)	1182 (18.2)	112 139 (16.5)	1943 (20.3)	1365 (17.2)
25–29	118 648 (16.3)	1502 (19.5)	2426 (17.8)	1204 (18.5)	110 720 (16.3)	1451 (15.1)	1344 (16.9)
30–34	101 231 (13.9)	1028 (13.3)	1972 (14.5)	972 (14.9)	94 871 (13.9)	1319 (13.8)	1069 (13.4)
35–39	97 084 (13.4)	933 (12.1)	1498 (11.0)	801 (12.3)	91 839 (13.5)	1110 (11.6)	904 (11.4)
40–44	81 054 (11.2)	607 (7.9)	1091 (8.0)	738 (11.3)	76 912 (11.3)	924 (9.6)	783 (9.8)
45–49	83 132 (11.4)	595 (7.7)	903 (6.6)	483 (7.4)	79 837 (11.7)	743 (7.7)	570 (7.2)
Highest education level attended							
No education	165 297 (22.8)	4245 (55.1)	2778 (20.4)	406 (6.2)	153 695 (22.6)	1545 (16.1)	2627 (33.0)
Primary	93 851 (12.9)	1547 (20.1)	3630 (26.7)	838 (12.9)	80 716 (11.9)	4020 (41.9)	3099 (38.9)
Secondary	360 744 (49.7)	1748 (22.7)	6158 (45.2)	4283 (65.8)	342 863 (50.4)	3622 (37.8)	2070 (26.0)
Higher	106 385 (14.6)	166 (2.2)	1049 (7.7)	980 (15.1)	103 618 (15.2)	409 (4.3)	162 (2.0)
Respondent currently working							
No	95 015 (13.1)	1872 (24.3)	5190 (38.1)	3678 (56.5)	75 702 (11.1)	2185 (22.8)	6389 (80.3)
Yes	51 925 (7.1)	5834 (75.7)	8426 (61.9)	2830 (43.5)	25 853 (3.8)	7412 (77.2)	1570 (19.7)
N.A.	579 338 (79.8)	0 (0.0)	0 (0.0)	0 (0.0)	579 337.9887 (85.1)	0 (0.0)	0 (0.0)
Current marital status							
Never in union	173 125 (23.8)	1900 (24.7)	4692 (34.5)	2655 (40.8)	159 298 (23.4)	2426 (25.3)	2154 (27.1)
Currently in union/living with a man	520 025 (71.6)	5409 (70.2)	7748 (56.9)	3194 (49.1)	492 756 (72.4)	5968 (62.2)	4949 (62.2)
Formerly in union/living with a man	33 127 (4.6)	396 (5.1)	1175 (8.6)	658 (10.1)	28 839 (4.2)	1203 (12.5)	856 (10.8)
Wealth index for urban/rural							
Poorest	136 872 (18.8)	1425 (18.5)	2385 (17.5)	1144 (17.6)	128 793 (18.9)	1641 (17.1)	1484 (18.7)
Poorer	146 914 (20.2)	1561 (20.3)	2578 (18.9)	1321 (20.3)	138 134 (20.3)	1786 (18.6)	1534 (19.3)
Middle	149 589 (20.6)	1551 (20.1)	2779 (20.4)	1357 (20.9)	140 354 (20.6)	1980 (20.6)	1568 (19.7)
Richer	149 025 (20.5)	1562 (20.3)	2898 (21.3)	1425 (21.9)	139 494 (20.5)	2039 (21.3)	1606 (20.2)
Richest	143 878 (19.8)	1607 (20.9)	2975 (21.9)	1260 (19.4)	134 117 (19.7)	2152 (22.4)	1766 (22.2)
Getting medical help for self							
No problem	291 262 (40.1)	0 (0.0)	0 (0.0)	0 (0.0)	291 262 (42.8)	0 (0.0)	0 (0.0)
Big problem	177 380 (24.4)	2404 (31.2)	5410 (39.7)	3291 (50.6)	159 666 (23.5)	3267 (34.1)	3341 (42.0)
Not a big problem	257 635 (35.5)	5302 (68.8)	8206 (60.3)	3216 (49.4)	229 965 (33.8)	6329 (66.0)	4618 (58.0)
Covered by health insurance							
No	515 250 (70.9)	7631 (99.0)	13 281 (97.5)	1893 (29.1)	475 922 (69.9)	9236 (96.2)	7286 (91.5)
Yes	211 027 (29.1)	74 (1.0)	334 (2.5)	4614 (70.9)	204 971 (30.1)	361 (3.8)	673 (8.5)
Use of internet							
Never	100 178 (13.8)	7158 (92.9)	9522 (69.9)	2022 (31.1)	68 341 (10.0)	8125 (84.7)	5011 (63.0)
Yes	46 762 (6.4)	548 (7.1)	4094 (30.1)	4485 (68.9)	33 215 (4.9)	1472 (15.3)	2948 (37.0)
N.A.	579 338 (79.8)	0 (0.0)	(0.0)	0 (0.0)	0 (85.1)	0 (0.0)	0 (0.0)

N.A., not available.

### Unmet needs level

In all six countries, unmet needs were highest under the NCD needs (i) criterion, reaching 99.0% (95% CI 99.0% to 99.0%), indicating nearly universal unmet needs. Under the looser NCD needs criterion (ii), unmet needs were 71.4% (95% CI 71.3% to 71.6%). In contrast, unmet needs for contraception, antenatal care and postnatal care were relatively lower, at 7.5% (95% CI 7.5% to 7.6%), 14.5% (95% CI 14.4% to 14.6%) and 14.5% (95% CI 14.3% to 14.6%), respectively (table 2).

**Table 2:** Unmet needs across countries

Indicator	Sample size, n	Unmet needs, n	Prevalence, % (95% CI)
Total			
NCD needs (i)	726 277	719 421	99.1 (99.0 to 99.1)
NCD needs (ii)	726 277	519 012	71.5 (71.3 to 71.6)
Contraception	726 277	55 043	7.6 (7.5 to 7.7)
Antenatal care	726 277	104 457	14.5 (14.4 to 14.6)
Postnatal care	726 277	105 338	14.5 (14.4 to 14.6)
Benin			
NCD needs (i)	7706	7687	99.8 (99.6 to 99.9)
NCD needs (ii)	7706	7096	92.1 (91.4 to 92.7)
Contraception	7706	2031	26.4 (25.3 to 27.4)
Antenatal care	7706	2737	35.5 (34.4 to 36.7)
Postnatal care	7706	3574	46.4 (45.2 to 47.6)
Cameroon			
NCD needs (i)	13 616	13 295	97.6 (97.3 to 97.9)
NCD needs (ii)	13 616	8901	65.4 (64.4 to 66.3)
Contraception	13 616	2403	17.7 (16.9 to 18.4)
Antenatal care	13 616	4404	32.3 (31.4 to 33.3)
Postnatal care	13 616	4739	34.8 (33.9 to 35.8)
Gabon			
NCD needs (i)	6507	6101	93.8 (92.7 to 94.7)
NCD needs (ii)	6507	4424	68.0 (66.1 to 69.9)
Contraception	6507	1642	25.2 (23.6 to 27.0)
Antenatal care	6507	1098	16.9 (15.5 to 18.3)
Postnatal care	6507	965	14.8 (13.5 to 16.3)
India			
NCD needs (i)	680 893	674 873	99.1 (99.1 to 99.2)
NCD needs (ii)	680 893	483 122	71.0 (70.8 to 71.1)
Contraception	680 893	46 137	6.8 (6.7 to 6.9)
Antenatal care	680 893	89 275	13.1 (13.0 to 13.2)
Postnatal care	680 893	89 683	13.2 (13.1 to 13.3)
Madagascar			
NCD needs (i)	9597	9532	99.3 (99.1 to 99.5)
NCD needs (ii)	9597	8878	92.5 (91.8 to 93.1)
Contraception	9597	1199	12.5 (11.8 to 13.3)
Antenatal care	9597	4104	42.8 (41.6 to 43.9)
Postnatal care	9597	3284	34.2 (33.1 to 35.3)
Mauritania			
NCD needs (i)	7959	7933	99.7 (99.4 to 99.8)
NCD needs (ii)	7959	6591	82.8 (81.8 to 83.8)
Contraception	7959	1631	20.5 (19.4 to 21.6)
Antenatal care	7959	2839	35.7 (34.4 to 37.0)
Postnatal care	7959	3093	38.9 (37.5 to 40.2)

NCD, non-communicable disease.

Significant variations in unmet needs were observed across countries. Benin had the highest unmet needs for NCD needs ((i) 99.7%), contraception (26.3%) and postnatal care (46.3%); Madagascar had the highest unmet needs for NCD needs ((ii) 92.5%) and antenatal care (42.7%); India had the lowest unmet needs across all SRH indicators: contraception (6.7%), antenatal care (13.1%) and postnatal care (13.1%). Gabon has significantly lower unmet needs for prenatal care (16.8%, 95% CI 15.5% to 18.3%) and postnatal care (14.8%, 95% CI 13.4% to 16.2%) compared with other African countries included in the study. Detailed levels of unmet needs by country are presented in table 2.

Unmet needs showed disparity across the three NCD screening ways. Table 3 showed the proportion of unmet needs for blood pressure measurement, blood glucose measurement and cervical cancer test in six countries. Among the total sample of 726 277 participants, unmet needs were most prevalent for cervical cancer testing, with 98.5% (95% CI 98.4% to 98.5%) of participants reporting they had never been tested, followed by blood glucose measurement (70.0%, 95% CI 69.8% to 70.1%) and blood pressure measurement (36.6%, 95% CI 36.4% to 36.7%). Across all countries, the unmet needs for cervical cancer screening are significantly higher than those for blood pressure measurement, ranging from 1.9 (Mauritania) to 3.1 (India) times higher. Furthermore, Gabon exhibits the lowest level of unmet needs for cervical screening, with a rate of 84.7% (95% CI 83.14% to 86.17%), which is more than 10 percentage points lower than that of other countries.

**Table 3:** Unmet needs for NCD management across countries

Indicator	Sample size, n	Unmet needs, n	Prevalence, % (95% CI)
Total			
Never had blood pressure measured	726 277	265 836	36.6 (36.5 to 36.8)
Never had blood glucose measured	726 277	508 428	70.0 (69.9 to 70.2)
Never tested for cervical cancer	726 277	715 485	98.5 (98.5 to 98.6)
Benin			
Never had blood pressure measured	7706	2970	38.5 (37.4 to 39.7)
Never had blood glucose measured	7706	7083	91.9 (91.2 to 92.5)
Never tested for cervical cancer	7706	7667	99.5 (99.3 to 99.6)
Cameroon			
Never had blood pressure measured	13 616	4359	32.0 (31.1 to 32.9)
Never had blood glucose measured	13 616	8919	65.5 (64.6 to 66.4)
Never tested for cervical cancer	13 616	13 145	96.5 (96.2 to 96.9)
Gabon			
Never had blood pressure measured	6507	1772	27.2 (25.5 to 29.0)
Never had blood glucose measured	6507	4794	73.7 (71.8 to 75.4)
Never tested for cervical cancer	6507	5513	84.7 (83.1 to 86.2)
India			
Never had blood pressure measured	680 893	248 783	36.5 (36.4 to 36.7)
Never had blood glucose measured	680 893	472 310	69.4 (69.2 to 69.5)
Never tested for cervical cancer	680 893	671 767	98.7 (98.6 to 98.7)
Madagascar			
Never had blood pressure measured	9597	3674	38.3 (37.2 to 39.4)
Never had blood glucose measured	9597	8869	92.4 (91.7 to 93.0)
Never tested for cervical cancer	9597	9472	98.7 (98.4 to 98.9)
Mauritania			
Never had blood pressure measured	7959	4280	53.8 (52.4 to 55.1)
Never had blood glucose measured	7959	6453	81.1 (80.0 to 82.1)
Never tested for cervical cancer	7959	7922	99.5 (99.3 to 99.7)

NCD, non-communicable disease.

### Unmet needs inequality

**Figure 1:**
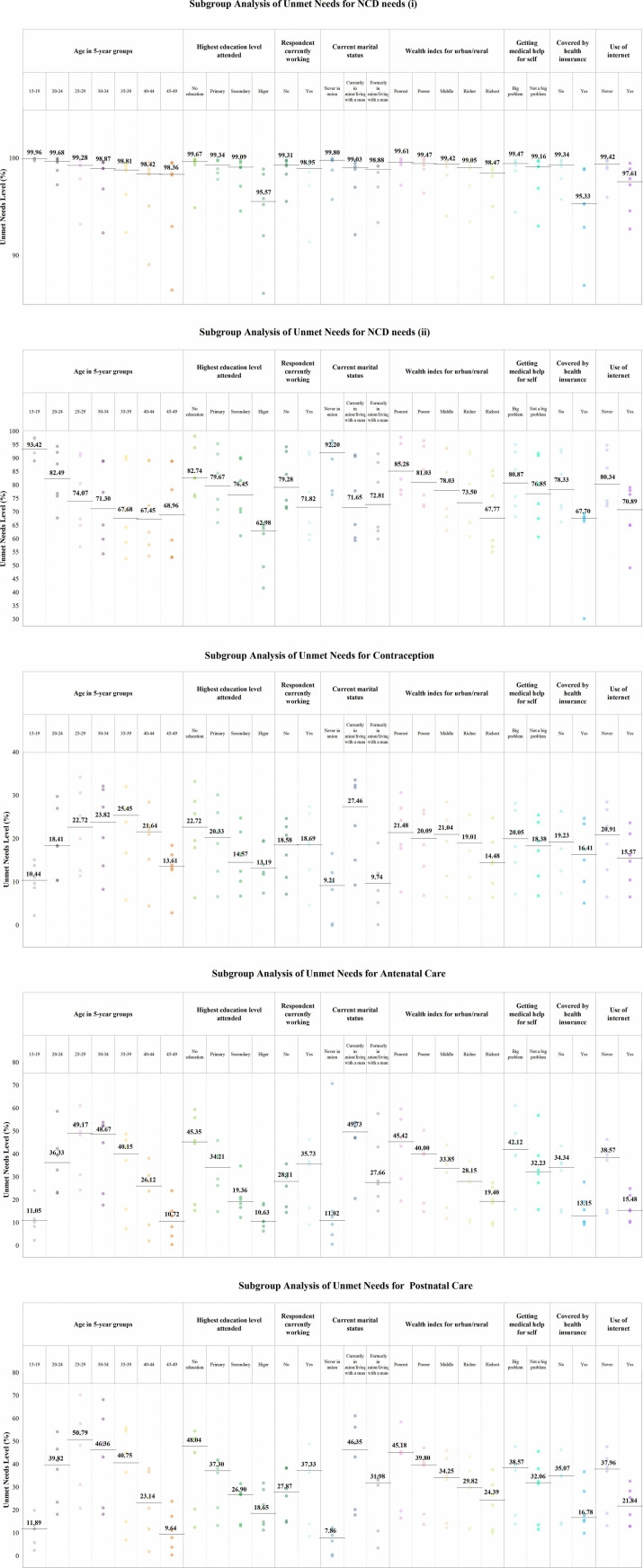
Subgroup analysis of unmet needs for NCD needs (i), NCD needs (ii), contraception, antenatal care and postnatal care across countries. Note: the figures are arranged in the following order: unmet needs for NCD needs (i), NCD needs (ii), contraception, antenatal care and postnatal care across countries. The median values are indicated by markers in the graphs. NCD, non-communicable disease.

Table 4 and figure 1 showed the unmet medication needs level by sociodemographic strata. Across all health domains, women with higher education levels, better economic status, easier access to healthcare, health insurance coverage and internet usage had lower proportions of unmet needs. This trend was particularly evident in antenatal and postnatal care. Individuals without formal education had significantly higher unmet needs compared with those with higher education, with unmet needs for antenatal care at 45.3% vs 10.6% (OR 4.3) and for postnatal care at 48.0% vs 18.6% (OR 2.6). Similarly, those in the poorest wealth quintile had higher unmet needs for antenatal care (45.4%) compared with the richest quintile (19.4%, OR 2.3), and for postnatal care (45.1% vs 24.3%, OR 1.9). The lack of health insurance also contributed to higher unmet needs, with those without insurance having higher unmet needs for antenatal care (34.3%) compared with those with insurance (13.1%, OR 2.6) and for postnatal care (35.0% vs 16.7%, OR 2.1). Additionally, individuals who had never used the internet had higher unmet needs for antenatal care (38.5%) compared with those who had used the internet (15.4%, OR 2.5) and for postnatal care (37.9% vs 21.8%, OR 1.7).

**Table 4:** Subgroup analysis for unmet needs in total population

Subgroups	NCD needs (i)	NCD needs (ii)	Contraception	Antenatal care	Postnatal care
	Unmet needs (N, %, 95% CI)	Unmet needs (N, %, 95% CI)	Unmet needs (N, %, 95% CI)	Unmet needs (N, %, 95% CI)	Unmet needs (N, %, 95% CI)
Age in 5-year groups					
15–19	124 552 (99.9, 99.9 to 99.9)	114 835 (92.1, 91.9 to 92.3)	3759 (3.0, 2.9 to 3.1)	4271 (3.4, 3.3 to 3.6)	4270 (3.4, 3.3 to 3.6)
20–24	119 960 (99.6, 99.5 to 99.6)	93 252 (77.4, 77.1 to 77.7)	13 334 (11.1, 10.8 to 11.3)	29 728 24.7, 24.3 to 25.0)	29 826 (24.8, 24.4 to 25.1)
25–29	117 686 (99.2, 99.1 to 99.3)	80 707 (68.0, 67.6 to 68.4)	14 614 (12.3, 12.1 to 12.6)	38 283 (32.3, 31.9 to 32.6)	38 725 (32.6, 32.3 to 33.0)
30–34	100 090 (98.9, 98.8 to 99.0)	66 450 (65.6, 65.2 to 66.1)	9419 (9.3, 9.1 to 9.6)	19 816 (19.6, 19.2 to 19.9)	20 417 (20.2, 19.8 to 20.5)
35–39	95 776 (98.7, 98.5 to 98.8)	62 835 (64.7, 64.3 to 65.1)	6762 (7.0, 6.7 to 7.2)	8770 (9.0, 8.8 to 9.3)	8724 (9.0, 8.7 to 9.2)
40–44	79 732 (98.4, 98.2 to 98.5)	50 997 (62.9, 62.4 to 63.4)	4345 (5.4, 5.2 to 5.6)	2724 (3.4, 3.2 to 3.5)	2566 (3.2, 3.0 to 3.3)
45–49	81 624 (98.2, 98.0 to 98.3)	49 935 (60.1, 59.6 to 60.5)	2809 (3.4, 3.2 to 3.5)	864 (1.0, 1.0 to 1.1)	810 (1.0, 0.9 to 1.1)
P value	<0.001	<0.001	<0.001	<0.001	<0.001
Highest education level attended					
No education	164 231 (99.4, 99.3 to 99.4)	127 782 (77.3, 77.0 to 77.6)	12 695 (7.7, 7.5 to 7.8)	29 424 (17.8, 17.6 to 18.0)	25 166 (15.2, 15.0 to 15.4)
Primary	92 898 (99.0, 98.9 to 99.1)	68 172 (72.6, 72.2 to 73.0)	7907 (8.4, 8.2 to 8.7)	17 250 (18.4, 18.1 to 18.7)	15 788 (16.8, 16.5 to 17.1)
Secondary	357 292 (99.0, 99.0 to 99.1)	257 393 (71.4, 71.1 to 71.6)	26 263 (7.3, 7.2 to 7.4)	46 474 (12.9, 12.7 to 13.0)	49 574 (13.7, 13.6 to 13.9)
Higher	104 999 (98.7, 98.6 to 98.8)	65 664 (61.7, 61.3 to 62.2)	8178 (7.7, 7.4 to 7.9)	11 309 (10.6, 10.4 to 10.9)	14 809 (13.9, 13.6 to 14.3)
P value	<0.001	<0.001	<0.001	<0.001	<0.001
Respondent currently working*					
No	94 156 (99.1, 99.0 to 99.2)	70 148 (73.8, 73.4 to 74.2)	9224 (9.7, 9.5 to 10.0)	16 575 (17.4, 17.1 to 17.8)	12 060 (23.2, 22.8 to 23.7)
Yes	51 005 (98.2, 98.1 to 98.4)	37 367 (72.0, 71.4 to 72.5)	6357 (12.2, 11.9 to 12.6)	12 056 (23.2, 22.8 to 23.7)	76 275 (13.2, 13.0 to 13.3)
P value	<0.001	<0.001	<0.001	<0.001	<0.001
Current marital status					
Never in union	172 771 (99.8, 99.8 to 99.8)	154 841 (89.4, 89.2 to 89.6)	1372 (0.8, 0.7 to 0.8)	1358 (0.8, 0.7 to 0.8)	1331 (0.8, 0.7 to 0.8)
Currently in union/living with a man	514 029 (98.8, 98.8 to 98.9)	342 722 (65.9, 65.7 to 66.1)	53 170 (10.2, 10.1 to 10.3)	100 728 (19.4, 19.2 to 19.5)	101 734 (19.6, 19.4 to 19.7)
Formerly in union/living with a man	32 621 (98.5, 98.3 to 98.7)	21 447 (64.7, 64.0 to 65.5)	502 (1.5, 1.4 to 1.7)	2371 (7.2, 6.8 to 7.5)	2273 (6.9, 6.5 to 7.2)
P value	<0.001	<0.001	<0.001	<0.001	<0.001
Wealth index for urban/rural					
Poorest	136 208 (99.5, 99.5 to 99.6)	112 983 (82.5, 82.3 to 82.8)	11 725 (8.6, 8.4 to 8.8)	29 184 (21.3, 21.0 to 21.6)	24 939 (18.2, 17.9 to 18.5)
Poorer	145 832 (99.3, 99.2 to 99.3)	112 809 (76.8, 76.5 to 77.1)	11 375 (7.7, 7.6 to 7.9)	24 068 (16.4, 16.1 to 16.6)	22 504 (15.3, 15.1 to 15.6)
Middle	148 262 (99.1, 99.0 to 99.2)	107 267 (71.7, 71.4 to 72.0)	11 130 (7.4, 7.3 to 7.6)	19 952 (13.3, 13.1 to 13.6)	20 523 (13.7, 13.5 to 14.0)
Richer	147 308 (98.8, 98.8 to 98.9)	99 282 (66.6, 66.3 to 67.0)	10 600 (7.1, 6.9 to 7.3)	17 039 (11.4, 11.2 to 11.7)	19 434 (13.0, 12.8 to 13.3)
Richest	141 810 (98.6, 98.5 to 98.7)	86 671 (60.2, 59.9 to 60.6)	10 212 (7.1, 6.9 to 7.3)	14 214 (9.9, 9.7 to 10.1)	17 936 (12.5, 12.2 to 12.7)
P value	<0.001	<0.001	<0.001	<0.001	<0.001
Getting medical help for self					
No problem	288 290 (99.0, 98.9 to 99.0)	193 900 (66.6, 66.3 to 66.8)	18 852 (6.5, 6.3 to 6.6)	31 272 (10.7, 10.6 to 10.9)	35 866 (12.3, 12.1 to 12.5)
Big problem	176 004 (99.2, 99.2 to 99.3)	135 463 (76.4, 76.1 to 76.6)	15 334 (8.6, 8.5 to 8.8)	31 727 (17.9, 17.7 to 18.1)	28 840 (16.3, 16.0 to 16.5)
Not a big problem	255 126 (99.0, 99.0 to 99.1)	189 648 (73.6, 73.4 to 73.8)	20 856 (8.1, 8.0 to 8.2)	41 458 (16.1, 15.9 to 16.3)	40 632 (15.8, 15.6 to 16.0)
P value	<0.001	<0.001	<0.001	<0.001	<0.001
Covered by health insurance					
No	510 961 (99.2, 99.1 to 99.2)	377 053 (73.2, 73.0 to 73.4)	43 103 (8.4, 8.3 to 8.5)	84 393 (16.4, 16.2 to 16.5)	83 721 (16.2, 16.1 to 16.4)
Yes	208 459 (98.8, 98.7 to 98.9)	141 958 (67.3, 67.0 to 67.5)	11 940 (5.7, 5.5 to 5.8)	20 064 (9.5, 9.4 to 9.7)	21 617 (10.2, 10.1 to 10.4)
P value	<0.001	<0.001	<0.001	<0.001	<0.001
Use of internet*					
Never	99 371 (99.2, 99.1 to 99.3)	77 086 (76.9, 76.6 to 77.3)	10 970 (11.0, 10.7 to 11.2)	22 651 (22.6, 22.3 to 22.9)	21 728 (21.7, 21.4 to 22.0)
Yes	45 789 (97.9, 97.7 to 98.1)	30 429 (65.1, 64.4 to 65.7)	4610 (9.9, 9.5 to 10.2)	5980 (12.8, 12.4 to 13.2)	7335 (15.7, 15.2 to 16.2)
P value	<0.001	<0.001	<0.001	<0.001	<0.001

* Represents part of sample failed to record the subgroup information.

NCD, non-communicable disease.

Employment status exerts distinct influences on unmet needs. Specifically, employed women have higher unmet needs levels for antenatal and postnatal care than unemployed women (antenatal care: 35.7% vs 28.1%; postnatal care: 37.3% vs 27.8%), but lower unmet needs levels for NCD prevention (NCD needs (i): employed 98.9% vs unemployed 99.3%; NCD needs (ii): employed 71.8% vs unemployed 79.2%). These findings will be further analysed and discussed in the Discussion section.

Demographic and socioeconomic factors significantly influenced the unmet needs for specific health screenings. Subgroup analyses for the specific NCD screenings are detailed in online supplemental S2–S4 tables, while the comprehensive subgroup results for all outcome variables, including the composite NCD measures (online supplemental S5 and S6 tables) and SRH services (online supplemental S7–S9 tables), are provided in the online supplemental tables. As shown in online supplemental S2–S4 figures, unmet needs were consistently higher among disadvantaged groups. For instance, individuals with no formal education had roughly twice the unmet need for blood pressure measurement (39.2% vs 19.0%) and 1.33 times that for blood glucose measurement (82.1% vs 61.7%) compared with those with higher education. Similarly, the unmet needs for cervical cancer screening were substantial across all population groups, with an overall rate remaining above 90%, although intergroup differences were observable. In general, individuals with higher education levels, better economic conditions, health insurance coverage and internet access consistently exhibited lower proportions of unmet needs for these screenings.

## Discussion

On a global scale, women of reproductive age are confronted with substantial health challenges, particularly in LMICs, where access to fundamental health services remains disparate. This study sheds light on the unmet needs for NCD management as well as the SRH healthcare, especially the highest unmet needs for cervical cancer screening in the six LMICs. The assessment of the socioeconomic gradient of these unmet needs among women of reproductive age revealed that populations with higher education levels, better economic status, easier access to healthcare, health insurance coverage and internet usage were more likely to have access to essential healthcare services, including both NCD prevention and SRH services. Furthermore, factors such as marital status, age and employment status exhibited differential impacts on the level of unmet needs for NCD prevention and SRH services.

The findings of the study demonstrate that there are significant gaps in the unmet needs for SRH services and NCDs across the six countries. Specifically, nearly universal unmet needs were observed under the strict NCD needs criterion ((i) 99.0%), while substantial gaps remained under the NCD needs criterion ((ii) 71.4%). The high unmet need for preventing NCDs in women has also been addressed by other studies, although with differences in definitions and observations.^27 37^ In the present study, the exceptionally high unmet needs for NCDs were primarily attributed to the significant shortfall in cervical cancer screening, with the unmet needs level reaching as high as 98.5% across the six countries. These findings are consistent with the results of other studies, such as one that found that only 9.6% of participants in the Philippines had undergone cervical cancer screening.^22^ In comparison, unmet needs for contraception, antenatal and postnatal care were comparatively lower (7.5%, 14.5% and 14.5%, respectively). However, the 7.5% prevalence of unmet contraceptive needs must be interpreted beyond its numerical value. In contexts of extremely low fertility rates and persistently high maternal mortality, this figure represents a substantial number of women who are denied the fundamental right to make autonomous decisions about their reproduction. The inability to access contraception not only constrains personal agency but also exacerbates risks linked to unintended pregnancies, often compounded by inadequate antenatal and postnatal care. This situation directly threatens women’s health and survival, underscoring an urgent need to situate service gaps within the broader framework of sexual and reproductive rights, in alignment with WHO’s EMPOWER(women have the power of dicision-making power and abilty to exercise their choices) strategy principles that emphasize empowering women and integrating maternal andnewborn care.^38^

At the country-specific level, India exhibited the lowest unmet needs across all SRH indicators: contraception (6.7%), antenatal care (13.1%) and postnatal care (13.1%). This relatively favourable performance can be attributed to several factors. First, India is the only Asian country in this comparison, which may inherently influence its healthcare infrastructure and resource allocation. Additionally, India has a higher density of medical professionals compared with the other countries, particularly nurses and midwives, compared with other countries. The nurse practitioner midwife programme, initiated in 2018, has played a pivotal role in enhancing the quality of care for women and newborns. Midwives were trained according to the International Confederation of Midwives standards, ensuring high-quality, dignified and respectful care.^39^ Despite the overall lower unmet needs level in India compared with other countries, regional disparities in the allocation of SRH services persist. Remote and impoverished areas may still lack sufficient healthcare facilities and specialised medical personnel to provide adequate prenatal care services.^40^ Future research should focus on exploring the internal inequities in unmet needs within countries to better understand and address these issues. Additionally, our study found that Gabon exhibited significantly lower unmet needs for antenatal care (16.8%, 95% CI 15.5% to 18.3%) and postnatal care (14.8%, 95% CI 13.4% to 16.2%). This is particularly noteworthy when compared with other African countries included in the study, such as Cameroon, where the unmet need for antenatal care was 32.3% (95% CI 31.4% to 33.2%), and 34.8% (95% CI 33.8% to 35.7%), respectively, and Benin, where the unmet need for antenatal care was 35.5% (95% CI 34.3% to 36.6%) and 46.3% (95% CI 45.1% to 47.5%). The establishment of the Central African Network for Women’s Reproductive Health (GCG) in 2009 is a significant development in the field of postabortion care. The primary objective of the GCG was to identify and overcome barriers to postabortion care in Gabon, with a particular emphasis on training midwives in manual vacuum aspiration and other relevant protocols. The GCG’s efforts have yielded notable outcomes, as evidenced by the remarkable results achieved.^41^ Conversely, the development of nursing personnel in other countries has been comparatively sluggish. In contrast, Madagascar reported the highest unmet need for antenatal care (42.8%, 95% CI 41.6% to 43.9%). It is important to note that the data for Madagascar were collected in 2021, during the COVID-19 pandemic, which significantly disrupted health service delivery. A recent study examining the impact of the pandemic on antenatal care in Madagascar compared data from 2019 (prepandemic) and 2021 (during the pandemic) and found a marked decline in service utilisation. Specifically, the proportion of women receiving no antenatal care increased from 11.9% to 15.8%,^42^ underscoring the pandemic’s adverse effects on maternal health service coverage and highlighting the need for resilient health systems.

Regarding NCD prevention, blood pressure measurement had the lowest overall unmet need (36.6%, 95% CI 36.5% to 36.8%), likely because it is a basic vital sign often integrated into primary healthcare contacts. However, substantial cross-country variation exists. Gabon and Cameroon are among the better-performing countries, possibly due to their relatively stable primary care systems and efforts to incorporate hypertension screening into routine practice. For example, Cameroon has implemented projects aimed at strengthening primary care services,^43^ which may have indirectly improved access to basic diagnostic services. In contrast, Mauritania had the highest unmet need (53.8%, 95% CI 52.4% to 55.1%), with more than half of women never having had their blood pressure measured. A World Bank report confirms that Mauritania’s primary healthcare system faces multiple challenges, including limited capacity for NCD services, shortages of basic diagnostic equipment and uneven distribution of human resources.^44^

Unmet need for blood glucose testing was substantially higher overall (70.0%, 95% CI 69.9% to 70.1%) than for blood pressure measurement. Cameroon (65.5%) and India (69.4%, 95% CI 69.2% to 69.5%) performed relatively well. India, for instance, has implemented national programmes such as the National Programme for Prevention and Control of Cancer, Diabetes, Cardiovascular Diseases and Stroke, which has helped raise public awareness and expand screening—though coverage and quality remain uneven.^45^ In contrast, Benin (91.9%, 95% CI 91.2% to 92.6%) and Madagascar (92.4%, 95% CI 91.8% to 93.0%) exhibited alarming rates, with over 90% of women never having undergone blood glucose testing. This reflects systemic barriers: glucose testing often requires more complex equipment (eg, glucometers and test strips), stable supply chains and trained personnel—resources that are frequently scarce in low-resource settings.^46^ The already critical situation in Madagascar may have been further exacerbated by the COVID-19 pandemic, which likely disrupted the supply chains for essential diagnostics and diverted limited health resources towards emergency response, thereby reducing the availability of routine NCD screening services.

Furthermore, Gabon exhibits the lowest unmet needs level for cervical screening at 84.7% (95% CI 83.1% to 86.1%), which is more than 10 percentage points lower than in the other five countries. This relatively high rate of cervical screening in Gabon can be attributed to the country’s explicit and proactive screening policies. The official recommendation in Gabon is to screen women aged 25–65 years every 3 years. This shorter screening interval facilitates the early detection of precancerous cervical lesions, thereby improving the cure rate. In comparison, India has a screening interval of 5 years,^47^ while Madagascar has screening recommendations but no specified interval, while Mauritania, Cameroon and Benin lack official screening recommendations altogether.^48^,^52^

The findings of the present study indicate that higher education levels, improved economic status, greater access to healthcare services, health insurance coverage and increased internet usage are associated with a reduced unmet need level across all health domains, including both for NCD and for SRH. These findings have been well-documented by previous scholars.^2122 31 53^,^55^ In order to provide more effective assistance to vulnerable populations in accessing services for the prevention of NCDs and SRH, certain countries have implemented targeted measures. For instance, the government of the Philippines launched the 5-year Philippine Primary Care Studies project to strengthen primary healthcare systems in rural and remote areas. This initiative included training workshops for nurses, midwives and village health workers, covering modules on essential intrapartum and newborn care, integrated management of childhood illness, NCDs and geriatrics. The project led to a substantial enhancement in the knowledge levels of these health workers.^56^ In China, the State Council promulgated the National Program for Women’s Development and the National Program for Children’s Development. These programmes integrated women’s and children’s health into the overarching national economic and social development strategy. With respect to insurance coverage, China implemented the New Rural Cooperative Medical System, which covered nearly the entire rural population and provided a range of health benefits for pregnant and maternity women. Furthermore, the Chinese government instituted a 9 year compulsory education system, thereby ensuring education coverage.^57^ These measures have been shown to have a significant impact on the health levels of vulnerable women in low socioeconomic status, leading to a notable decrease in the maternal mortality rate from 53 per 100 000 in 2000^58^ to 15.1 per 100 000 in 2023.^59^ The Value-Based Care model has emerged as a particularly effective solution for populations with a lower wealth index. The fundamental objective of the Value-Based Care model is to curtail patients’ medical expenditures by means of preventive care, chronic disease management and the reduction of superfluous medical services.^60^ In the USA, the Value-Based Care models such as the Medicare Shared Savings Program, Chronic Care Management and Transitional Care Management provide continuous care support, helping patients manage their health conditions and reduce unnecessary medical costs.^61^ For instance, the Medicare Shared Savings Program has been instrumental in achieving cost savings of over US$1.8 billion for Medicare beneficiaries in 2020.^62^ It is noteworthy that other countries can draw valuable insights from these successful experiences and implement similar measures to enhance vulnerable populations’ access to NCD prevention and SRH services. By fortifying primary health systems, expanding health insurance coverage and implementing compulsory education, these nations can effectively address unmet health needs and enhance health outcomes for vulnerable populations.

The findings of this study indicated that the impact of employment on the unmet needs level, NCD prevention and SRH services exhibited a divergence in outcomes. In the context of NCD prevention, the data indicated that employed women exhibited a lower prevalence of unmet needs under both NCD needs definitions ((i) 98.9% and (ii) 71.8%), in comparison to unemployed women ((i) 99.3% and (ii) 79.2%). This discrepancy could be attributed, at least in part, to the higher propensity of employed women to engage in cervical cancer screening.^22^ This phenomenon could be associated with an increased level of health consciousness or greater accessibility to healthcare services through employment. However, a contrasting trend emerges when examining SRH services, where employed women exhibited higher unmet needs for both antenatal (35.7% compared with 48.6%) and postnatal care (37.3% compared with 46.3%). This discrepancy may be indicative of the constraints imposed by employment, which could hinder access to essential healthcare services despite the increased autonomy and resources that come with employment.^63^ This discrepancy underscores the necessity for healthcare systems that are attuned to the unique challenges faced by employed women in accessing SRH services, particularly in the context of antenatal and postnatal care.

In light of these findings, governments and non-governmental organisations should prioritise investment in prevention services for NCDs, particularly in LMICs. National guidelines must delineate target populations for screening and vaccination, specify screening intervals and recommend detection methods or tools. Addressing the critical disparity in cervical cancer screening, a systematic, nationwide programme for cervical cancer screening should be implemented to ensure regular screening for all women of childbearing age. Research has confirmed that integrating cervical cancer screening into existing family planning clinics is a potentially effective strategy, given that family planning services are more accessible and frequently used by the general population.^64^ Furthermore, there is a need for substantial investment in training prenatal and postnatal care providers to enhance their professional skills and service quality, particularly with regard to emergency skills training.^65^ The Philippine Primary Care Studies project exemplifies a model that other nations may wish to emulate. By enhancing working conditions and remuneration for care providers, more talented individuals can be attracted to this field, thereby enhancing the efficiency and quality of the entire healthcare system.^66^ The present study identified vulnerable populations as a priority for intervention. These populations include individuals with low education levels, poor economic status, limited access to health resources, lack of health insurance and insufficient internet usage. These populations face greater difficulties in accessing basic health services. Efforts should be made to promote long-term care insurance coverage, which has been confirmed to improve women’s health status, including decreasing the risk of NCDs.^67^ Moreover, governments can benefit from China’s experience by expanding health insurance coverage through programmes such as the New Rural Cooperative Medical System, ensuring that vulnerable women of reproductive age have access to a range of health benefits. The integration of telehealth and mobile health technologies to facilitate health education, remote consultation and appointment services can enhance the accessibility and utilisation of health services for women residing in remote areas, thereby improving their access to medical resources.^68^ The implementation of compulsory education and the adoption of a viable Value-Based Care model, as previously highlighted, are of paramount importance in addressing the health disparities faced by vulnerable populations. The employment status of women of childbearing age has been shown to have a significant impact on the unmet needs for NCDs and SRH, underscoring the need for governments and policymakers to direct greater attention to the challenges faced by employed women in accessing these services. Concomitantly, the promotion of gender equality through global cooperation, policy support and investment in scientific research is imperative. This objective necessitates the reduction of gender disparities in social, economic and health fields, as well as the assurance of women’s full participation in decision-making in science and health.^69^ Empathy and understanding of women’s challenges among female learners and decision-makers are crucial factors in this regard. Addressing gender inequality, which has profound negative impacts on women’s health worldwide, is another critical factor. This includes higher maternal mortality, gender-based violence and underrepresentation in scientific and health sectors, which are particularly pronounced in LMICs and against the backdrop of racial inequality.^6 20^

This study has several strengths. First, it encompasses a substantial and diverse sample of women of reproductive age from six nationally representative surveys that had full target data from the DHS. Second, the study employed two distinct definitions of NCDs, a methodological approach that enhanced the robustness of the findings. Third, unmet needs of reproductive-age women concerning NCDs and SRH were compared, and demographic and socioeconomic factors were analysed to identify vulnerable populations. However, the study is not without limitations. First, due to data constraints, we were unable to capture specific reasons for unmet needs or instances of foregone healthcare. Additionally, the number of countries included in the analysis was limited, although all the nationally representative surveys have been included for analysis that had full target data from the DHS. Third, we acknowledge that the definition of unmet needs for breast cancer may not include women aged 25–64 recommended by the WHO due to the age restrictions in DHS data, thus weakening the representativeness. Future surveys should aim to capture detailed reasons for unmet needs and include a broader range of LMICs to achieve the generalisability of the findings.

## Conclusion

In conclusion, our study underscores the necessity for tailored interventions to address the high unmet need for the prevention of NCDs, particularly cervical cancer screening, among women of reproductive age in LMICs. The WHO has set a goal to eliminate cervical cancer, aiming to reduce the incidence rate to fewer than 4 cases per 1 00 000 women by 2030.^70^ Achieving this objective necessitates the implementation of strategic actions aimed at enhancing access to essential health services. Furthermore, our study identified vulnerable populations in terms of service utilisation and the varying impacts of different social and demographic factors on the unmet need for NCD prevention and SRH services. These findings underscore the need for concerted action by relevant authorities and the implementation of purposeful measures. Collective and impactful initiatives are imperative for ensuring women’s well-being throughout their lives, and they are pivotal for the realisation of Universal Health Coverage and the achievement of the Sustainable Development Goals by the year 2030.

## Supplementary material

10.1136/bmjopen-2025-105422 online supplemental file 1

## Data Availability

Data are available in a public, open access repository.
