# Supplementary material for: Unmet needs for non-communicable diseases and sexual and reproductive health services among women of reproductive age in low-and-middle-income countries: evidence from the Demographic and Health Surveys
Source: BMJ Open. 2026 Feb 9;16(2):e105422. doi: 10.1136/bmjopen-2025-105422 (PMC12887515; doi:10.1136/bmjopen-2025-105422)
Supplement: online supplemental file 1 [file bmjopen-16-2-s001.pdf]

Unmet Needs for Non-Communicable Diseases and Sexual and Reproductive Health Services among women of reproductive age in Low-And-Middle-Income Countries: Evidence from the Demographic and Health Surveys

Short title: Unmet Needs for NCDs and SRH Services in women in LMICs

Yishu Yin, Yeting Du, Zhi-Jie Zheng, Minghui Ren, Minmin Wang, Yinzi Jin

S1 Fig. The Sample screening process. Note: Sample weight is an eight-digit variable with six implied decimal places. To apply the sample weight, it must be divided by 1,000,000 before the weighting factor is utilized. All sample weights are normalized so that the weighted number of cases matches the unweighted number of cases when using the full dataset without any subsampling.

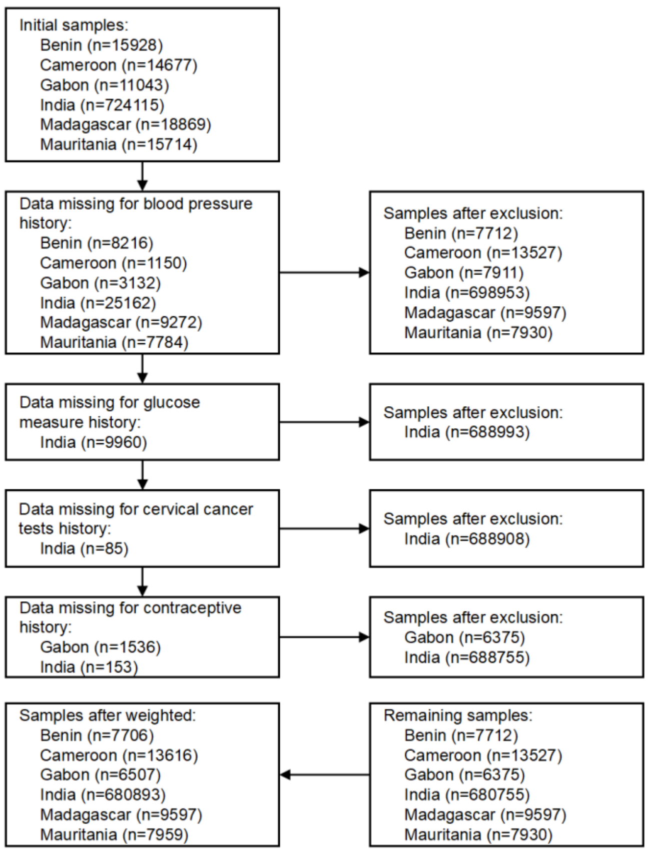

S1 Table. Details of questions used to determine participants' needs

Variable	Categories	Questions	Answers	Outcome
s1401	NCD needs	ever had blood pressure measured	0 no	unmet needs
			1 yes	met needs
			8 don't know	unmet needs
s1406	NCD needs	ever had blood sugar measured	0 no	unmet needs
			1 yes	met needs
			8 don't know	unmet needs
s1426	NCD needs	tested for cervical cancer	0 no	unmet needs
			1 yes	met needs
			8 don't know	unmet needs
v626a	Contraception	unmet need for contraception	0 never had sex	met needs
			1 unmet need for spacing	unmet needs
			2 unmet need for limiting	unmet needs
			3 using for spacing	met needs
			4 using for limiting	met needs
			7 no unmet need	met needs
			8 not married and no sex in last 30 days	met needs
			9 infecund, menopausal	met needs
m2n_1	Antenatal Care	prenatal: no one	0 no: some care	met needs

			1 yes: no care	unmet needs
m13_1	Antenatal Care	timing of 1st antenatal check (months)	≤3	met needs
			> 3	unmet needs
m14_1	Antenatal Care	number of antenatal visits during pregnancy	≥4	met needs
			< 4	unmet needs
m15_1	Antenatal Care	place of delivery	10 home	unmet needs
			11 respondent's home	unmet needs
			12 other home	unmet needs
			20 public sector	met needs
			21 government hospital	met needs
			22 cs govt health professional	met needs
			23 cs govt health professional	met needs
			26 other public sector	met needs
			30 private sector	met needs
			31 private hospital/clinic	met needs
			36 other private sector	met needs
			96 other	unmet needs
m70_1	Postnatal Care	baby postnatal check within 2 months	0 no	unmet needs
			1 yes	met needs
			8 don't know	unmet needs

S2 Table. Subgroup Analysis Among Countries: Unmet needs for Ever Had Blood Pressure Measured

	Sample Size	Unmet Needs (N, %)	95%CI	P value
**Benin**				
Age in 5--year groups				
15--19	1617	1575 (97.35)	(96.32,98.10)	<0.0001
20--24	1423	1308 (91.96)	(90.29,93.37)	
25--29	1502	1379 (91.82)	(90.23,93.18)	
30--34	1028	917 (89.19)	(86.91,91.11)	
35--39	933	837 (89.75)	(87.43,91.67)	
40--44	607	540 (88.89)	(85.92,91.29)	
45--49	595	527 (88.42)	(85.38,90.91)	
Highest education level attended				
No education	4245	3978 (93.72)	(92.88,94.47)	<0.0001
Primary	1547	1415 (91.46)	(89.78,92.88)	
Secondary	1748	1579 (90.34)	(88.77,91.70)	
Higer	166	111 (66.47)	(58.51,73.60)	
Respondent currently working				
No	1872	1760 (94.05)	(92.76,95.12)	<0.0001
Yes	5834	5322 (91.23)	(90.41,91.98)	
N.A.	0	0 (0.00)	--	
Current marital status				
Never in union	1900	1827 (96.15)	(95.08,96.99)	<0.0001
Currently in union/living with a man	5409	4907 (90.72)	(89.86,91.52)	
Formerly in union/living with a man	396	348 (87.87)	(83.77,91.05)	
Wealth index for urban/rural				
Poorest	1425	1357 (95.24)	(93.93,96.28)	<0.0001
Poorer	1561	1470 (94.19)	(92.80,95.33)	
Middle	1551	1447 (93.29)	(91.79,94.54)	
Richer	1562	1435 (91.89)	(90.21,93.29)	
Richest	1607	1373 (85.44)	(83.52,87.17)	
Getting medical help for self				
No problem	0	0 (0.00)	--	<0.0001
Big problem	2404	2214 (92.08)	(90.81,93.18)	
Not a big problem	5302	4869 (91.84)	(91.01,92.60)	
Covered by health insurance				
No	7631	7032 (92.14)	(91.46,92.77)	<0.0001
Yes	74	51 (68.07)	(56.22,77.97)	
Use of Internet				
Never	7158	6655 (92.97)	(92.30,93.59)	<0.0001
Yes	548	428 (78.09)	(74.23,81.51)	
N.A.	0	0 (0.00)	--	
**Cameroon**				
Age in 5--year groups				

15--19	3309	2932 (88.61)	(87.35,89.75)	<0.0001
20--24	2417	1618 (66.93)	(64.74,69.06)	
25--29	2426	1401 (57.76)	(55.41,60.09)	
30--34	1972	1081 (54.80)	(52.12,57.45)	
35--39	1498	807 (53.90)	(50.86,56.91)	
40--44	1091	595 (54.52)	(51.02,57.98)	
45--49	903	485 (53.68)	(49.84,57.49)	
Highest education level attended				
No education	2778	2294 (82.56)	(80.75,84.24)	<0.0001
Primary	3630	2426 (66.84)	(65.07,68.56)	
Secondary	6158	3756 (61.00)	(59.56,62.42)	
Higer	1049	442 (42.17)	(38.59,45.82)	
Respondent currently working				
No	5190	3686 (71.02)	(69.53,72.47)	<0.0001
Yes	8426	5233 (62.11)	(60.87,63.32)	
N.A.	0	0 (0.00)	--	
Current marital status				
Never in union	4692	3569 (76.06)	(74.64,77.42)	<0.0001
Currently in union/living with a man	7748	4631 (59.77)	(58.45,61.08)	
Formerly in union/living with a man	1175	719 (61.17)	(57.89,64.35)	
Wealth index for urban/rural				
Poorest	2385	1867 (78.29)	(76.24,80.20)	<0.0001
Poorer	2578	1866 (72.40)	(70.35,74.36)	
Middle	2779	1768 (63.63)	(61.45,65.75)	
Richer	2898	1764 (60.84)	(58.75,62.90)	
Richest	2975	1654 (55.58)	(53.48,57.65)	
Getting medical help for self				
No problem	0	0 (0.00)	--	<0.0001
Big problem	5410	3918 (72.43)	(71.00,73.81)	
Not a big problem	8206	5001 (60.94)	(59.68,62.18)	
Covered by health insurance				
No	13281	8813 (66.36)	(65.41,67.30)	<0.0001
Yes	334	105 (31.52)	(25.55,38.16)	
Use of internet				
Never	9522	6889 (72.35)	(71.29,73.39)	<0.0001
Yes	4094	2030 (49.58)	(47.75,51.42)	
N.A.	0	0 (0.00)	--	
**Gabon**				P value
Age in 5--year groups				
15--19	1127	1009 (89.48)	(86.33,91.97)	<0.0001
20--24	1182	939 (79.44)	(75.24,83.09)	
25--29	1204	848 (70.39)	(65.98,74.45)	
30--34	972	683 (70.24)	(65.27,74.78)	
35--39	801	531 (66.37)	(60.62,71.68)	
40--44	738	480 (65.03)	(58.35,71.17)	
45--49	483	305 (63.17)	(56.41,69.45)	
Highest education level attended				
No education	406	321 (79.00)	(70.77,85.38)	<0.0001
Primary	838	664 (79.16)	(75.02,82.78)	
Secondary	4283	3229 (75.38)	(73.20,77.44)	
Higer	980	582 (59.35)	(53.47,64.98)	
Respondent currently working				
No	3678	2915 (79.27)	(77.04,81.33)	<0.0001
Yes	2830	1879 (66.42)	(63.37,69.33)	
N.A.	0	0 (0.00)	--	
Current marital status				
Never in union	2655	2172 (81.82)	(79.15,84.21)	<0.0001
Currently in union/living with a man	3194	2139 (66.96)	(64.15,69.66)	
Formerly in union/living with a man	658	484 (73.45)	(68.18,78.13)	
Wealth index for urban/rural				
Poorest	1144	930 (81.25)	(78.29,83.89)	<0.0001
Poorer	1321	1027 (77.74)	(73.72,81.30)	
Middle	1357	1005 (74.02)	(69.84,77.81)	
Richer	1425	1021 (71.67)	(67.28,75.69)	
Richest	1260	812 (64.45)	(59.72,68.92)	
Getting medical help for self				

No problem	0	0 (0.00)	--	0.508
Big problem	3291	2405 (73.08)	(70.53,75.49)	
Not a big problem	3216	2389 (74.30)	(71.61,76.81)	
Covered by health insurance				
No	1893	1403 (74.15)	(70.57,77.42)	0.750
Yes	4614	3391 (73.49)	(71.33,75.55)	
Use of internet				
Never	2022	1602 (79.21)	(76.29,81.86)	<0.0001
Yes	4485	3193 (71.19)	(68.86,73.41)	
N.A.	0	0 (0.00)	--	
**India**				P value
Age in 5--year groups				
15--19	114575	103147 (90.03)	(89.78,90.26)	<0.0001
20--24	112139	84392 (75.26)	(74.90,75.61)	
25--29	110720	73141 (66.06)	(65.66,66.46)	
30--34	94871	60672 (63.95)	(63.51,64.39)	
35--39	91839	57727 (62.86)	(62.41,63.30)	
40--44	76912	46931 (61.02)	(60.53,61.51)	
45--49	79837	46300 (57.99)	(57.50,58.48)	
Highest education level attended				
No education	153695	115546 (75.18)	(74.89,75.47)	<0.0001
Primary	80716	56210 (69.64)	(69.19,70.09)	
Secondary	342863	238377 (69.53)	(69.30,69.75)	
Higer	103618	62178 (60.01)	(59.55,60.46)	
Respondent currently working				
No	75702	53353 (70.48)	(70.01,70.94)	<0.0001
Yes	25853	16720 (64.67)	(63.80,65.54)	
N.A.	579338	402237 (69.43)	(69.26,69.60)	
Current marital status				
Never in union	159298	139491 (87.57)	(87.33,87.79)	<0.0001
Currently in union/living with a man	492756	315065 (63.94)	(63.75,64.13)	
Formerly in union/living with a man	28839	17755 (61.57)	(60.74,62.39)	
Wealth index for urban/rural				
Poorest	128793	104190 (80.90)	(80.58,81.21)	<0.0001
Poorer	138134	103372 (74.83)	(74.50,75.16)	
Middle	140354	97773 (69.66)	(69.32,70.00)	
Richer	139494	89453 (64.13)	(63.77,64.48)	
Richest	134117	77521 (57.80)	(57.42,58.18)	
Getting medical help for self				
No problem	291262	189167 (64.95)	(64.69,65.20)	<0.0001
Big problem	159666	118541 (74.24)	(73.95,74.53)	
Not a big problem	229965	164602 (71.58)	(71.32,71.84)	
Covered by health insurance				
No	475922	337435 (70.90)	(70.71,71.09)	<0.0001
Yes	204971	134875 (65.80)	(65.52,66.08)	
Use of internet				
Never	68341	49011 (71.72)	(71.24,72.19)	<0.0001
Yes	33215	21062 (63.41)	(62.63,64.19)	
N.A.	579338	402237 (69.43)	(69.26,69.60)	
**Madagascar**				P value
Age in 5--year groups				
15--19	2106	2042 (96.93)	(95.96,97.68)	<0.0001
20--24	1943	1839 (94.64)	(93.32,95.71)	
25--29	1451	1320 (90.92)	(88.93,92.59)	
30--34	1319	1176 (89.15)	(86.89,91.05)	
35--39	1110	1007 (90.75)	(88.39,92.67)	
40--44	924	828 (89.58)	(86.91,91.76)	
45--49	743	658 (88.56)	(85.53,91.03)	
Highest education level attended				
No education	1545	1515 (98.01)	(97.11,98.64)	<0.0001
Primary	4020	3830 (95.28)	(94.41,96.03)	
Secondary	3622	3259 (89.97)	(88.70,91.11)	
Higer	409	265 (64.85)	(59.13,70.18)	
Respondent currently working				
No	2185	2007 (91.86)	(90.26,93.21)	0.373
Yes	7412	6862 (92.58)	(91.84,93.27)	

N.A.	0	0 (0.00)	--	
Current marital status				
Never in union	2426	2314 (95.38)	(94.32,96.25)	<0.0001
Currently in union/living with a man	5968	5447 (91.27)	(90.35,92.11)	
Formerly in union/living with a man	1203	1108 (92.14)	(90.04,93.83)	
Wealth index for urban/rural				
Poorest	1641	1605 (97.86)	(96.98,98.48)	<0.0001
Poorer	1786	1725 (96.59)	(95.55,97.40)	
Middle	1980	1852 (93.53)	(92.20,94.65)	
Richer	2039	1888 (92.59)	(91.18,93.79)	
Richest	2152	1799 (83.62)	(81.46,85.58)	
Getting medical help for self				
No problem	0	0 (0.00)	--	<0.0001
Big problem	3267	3115 (95.33)	(94.37,96.14)	
Not a big problem	6329	5754 (90.91)	(90.02,91.74)	
Covered by health insurance				
No	9236	8620 (93.33)	(92.68,93.92)	<0.0001
Yes	361	249 (69.14)	(63.09,74.59)	
Use of internet				
Never	8125	7702 (94.80)	(94.18,95.36)	<0.0001
Yes	1472	1167 (79.26)	(76.65,81.65)	
N.A.	0	0 (0.00)	--	
**Mauritania**				P value
Age in 5--year groups				
15--19	1922	1798 (93.50)	(91.91,94.80)	<0.0001
20--24	1365	1178 (86.24)	(83.87,88.32)	
25--29	1344	1071 (79.74)	(76.92,82.29)	
30--34	1069	807 (75.51)	(72.12,78.61)	
35--39	904	625 (69.07)	(65.09,72.79)	
40--44	783	544 (69.43)	(65.37,73.21)	
45--49	570	431 (75.51)	(71.16,79.40)	
Highest education level attended				
No education	2627	2144 (81.60)	(79.76,83.32)	<0.0001
Primary	3099	2560 (82.60)	(80.91,84.17)	
Secondary	2070	1647 (79.54)	(77.17,81.72)	
Higer	162	103 (63.29)	(52.60,72.82)	
Respondent currently working				
No	6389	5263 (82.38)	(81.19,83.50)	<0.0001
Yes	1570	1190 (75.81)	(73.04,78.38)	
N.A.	0	0 (0.00)	--	
Current marital status				
Never in union	2154	2006 (93.13)	(91.57,94.42)	<0.0001
Currently in union/living with a man	4949	3770 (76.18)	(74.68,77.62)	
Formerly in union/living with a man	856	677 (79.10)	(75.52,82.27)	
Wealth index for urban/rural				
Poorest	1484	1290 (86.94)	(84.64,88.94)	<0.0001
Poorer	1534	1294 (84.33)	(81.90,86.50)	
Middle	1568	1301 (82.94)	(80.44,85.18)	
Richer	1606	1256 (78.23)	(75.62,80.64)	
Richest	1766	1312 (74.27)	(71.71,76.68)	
Getting medical help for self				
No problem	0	0 (0.00)	--	<0.0001
Big problem	3341	2806 (84.00)	(82.40,85.47)	
Not a big problem	4618	3647 (78.97)	(77.46,80.40)	
Covered by health insurance				
No	7286	6013 (82.53)	(81.42,83.58)	<0.0001
Yes	673	440 (65.44)	(60.68,69.90)	
Use of internet				
Never	5011	4255 (84.91)	(83.68,86.06)	<0.0001
Yes	2948	2198 (74.57)	(72.48,76.55)	
N.A.	0	0 (0.00)	--	

S3 Table. Subgroup Analysis Among Countries: Unmet needs for Ever Had Blood Glucose Measured

	Sample Size	Unmet Needs (N, %)	95%CI	P value
**Benin**				

Age in 5--year groups				
15--19	1617	1575 (97.35)	(96.32,98.10)	<0.0001
20--24	1423	1308 (91.96)	(90.29,93.37)	
25--29	1502	1379 (91.82)	(90.23,93.18)	
30--34	1028	917 (89.19)	(86.91,91.11)	
35--39	933	837 (89.75)	(87.43,91.67)	
40--44	607	540 (88.89)	(85.92,91.29)	
45--49	595	527 (88.42)	(85.38,90.91)	
Highest education level attended				
No education	4245	3978 (93.72)	(92.88,94.47)	<0.0001
Primary	1547	1415 (91.46)	(89.78,92.88)	
Secondary	1748	1579 (90.34)	(88.77,91.70)	
Higer	166	111 (66.47)	(58.51,73.60)	
Respondent currently working				
No	1872	1760 (94.05)	(92.76,95.12)	<0.0001
Yes	5834	5322 (91.23)	(90.41,91.98)	
N.A.	0	0 (0.00)	--	
Current marital status				
Never in union	1900	1827 (96.15)	(95.08,96.99)	<0.0001
Currently in union/living with a man	5409	4907 (90.72)	(89.86,91.52)	
Formerly in union/living with a man	396	348 (87.87)	(83.77,91.05)	
Wealth index for urban/rural				
Poorest	1425	1357 (95.24)	(93.93,96.28)	<0.0001
Poorer	1561	1470 (94.19)	(92.80,95.33)	
Middle	1551	1447 (93.29)	(91.79,94.54)	
Richer	1562	1435 (91.89)	(90.21,93.29)	
Richest	1607	1373 (85.44)	(83.52,87.17)	
Getting medical help for self				
No problem	0	0 (0.00)	--	<0.0001
Big problem	2404	2214 (92.08)	(90.81,93.18)	
Not a big problem	5302	4869 (91.84)	(91.01,92.60)	
Covered by health insurance				
No	7631	7032 (92.14)	(91.46,92.77)	<0.0001
Yes	74	51 (68.07)	(56.22,77.97)	
Use of internet				
Never	7158	6655 (92.97)	(92.30,93.59)	<0.0001
Yes	548	428 (78.09)	(74.23,81.51)	
N.A.	0	0 (0.00)	--	
**Cameroon**				
Age in 5--year groups				
15--19	3309	2932 (88.61)	(87.35,89.75)	<0.0001
20--24	2417	1618 (66.93)	(64.74,69.06)	
25--29	2426	1401 (57.76)	(55.41,60.09)	
30--34	1972	1081 (54.80)	(52.12,57.45)	
35--39	1498	807 (53.90)	(50.86,56.91)	
40--44	1091	595 (54.52)	(51.02,57.98)	
45--49	903	485 (53.68)	(49.84,57.49)	
Highest education level attended				
No education	2778	2294 (82.56)	(80.75,84.24)	<0.0001
Primary	3630	2426 (66.84)	(65.07,68.56)	
Secondary	6158	3756 (61.00)	(59.56,62.42)	
Higer	1049	442 (42.17)	(38.59,45.82)	
Respondent currently working				
No	5190	3686 (71.02)	(69.53,72.47)	<0.0001
Yes	8426	5233 (62.11)	(60.87,63.32)	
N.A.	0	0 (0.00)	--	
Current marital status				
Never in union	4692	3569 (76.06)	(74.64,77.42)	<0.0001
Currently in union/living with a man	7748	4631 (59.77)	(58.45,61.08)	
Formerly in union/living with a man	1175	719 (61.17)	(57.89,64.35)	
Wealth index for urban/rural				
Poorest	2385	1867 (78.29)	(76.24,80.20)	<0.0001
Poorer	2578	1866 (72.40)	(70.35,74.36)	
Middle	2779	1768 (63.63)	(61.45,65.75)	
Richer	2898	1764 (60.84)	(58.75,62.90)	
Richest	2975	1654 (55.58)	(53.48,57.65)	

Getting medical help for self				
No problem	0	0 (0.00)	--	<0.0001
Big problem	5410	3918 (72.43)	(71.00,73.81)	
Not a big problem	8206	5001 (60.94)	(59.68,62.18)	
Covered by health insurance				
No	13281	8813 (66.36)	(65.41,67.30)	<0.0001
Yes	334	105 (31.52)	(25.55,38.16)	
Use of internet				
Never	9522	6889 (72.35)	(71.29,73.39)	<0.0001
Yes	4094	2030 (49.58)	(47.75,51.42)	
N.A.	0	0 (0.00)	--	
**Gabon**				P value
Age in 5--year groups				
15--19	1127	1009 (89.48)	(86.33,91.97)	<0.0001
20--24	1182	939 (79.44)	(75.24,83.09)	
25--29	1204	848 (70.39)	(65.98,74.45)	
30--34	972	683 (70.24)	(65.27,74.78)	
35--39	801	531 (66.37)	(60.62,71.68)	
40--44	738	480 (65.03)	(58.35,71.17)	
45--49	483	305 (63.17)	(56.41,69.45)	
Highest education level attended				
No education	406	321 (79.00)	(70.77,85.38)	<0.0001
Primary	838	664 (79.16)	(75.02,82.78)	
Secondary	4283	3229 (75.38)	(73.20,77.44)	
Higer	980	582 (59.35)	(53.47,64.98)	
Respondent currently working				
No	3678	2915 (79.27)	(77.04,81.33)	<0.0001
Yes	2830	1879 (66.42)	(63.37,69.33)	
N.A.	0	0 (0.00)	--	
Current marital status				
Never in union	2655	2172 (81.82)	(79.15,84.21)	<0.0001
Currently in union/living with a man	3194	2139 (66.96)	(64.15,69.66)	
Formerly in union/living with a man	658	484 (73.45)	(68.18,78.13)	
Wealth index for urban/rural				
Poorest	1144	930 (81.25)	(78.29,83.89)	<0.0001
Poorer	1321	1027 (77.74)	(73.72,81.30)	
Middle	1357	1005 (74.02)	(69.84,77.81)	
Richer	1425	1021 (71.67)	(67.28,75.69)	
Richest	1260	812 (64.45)	(59.72,68.92)	
Getting medical help for self				
No problem	0	0 (0.00)	--	0.508
Big problem	3291	2405 (73.08)	(70.53,75.49)	
Not a big problem	3216	2389 (74.30)	(71.61,76.81)	
Covered by health insurance				
No	1893	1403 (74.15)	(70.57,77.42)	0.750
Yes	4614	3391 (73.49)	(71.33,75.55)	
Use of internet				
Never	2022	1602 (79.21)	(76.29,81.86)	<0.0001
Yes	4485	3193 (71.19)	(68.86,73.41)	
on	0	0 (0.00)	--	
**India**				P value
Age in 5--year groups				
15--19	114575	103147 (90.03)	(89.78,90.26)	<0.0001
20--24	112139	84392 (75.26)	(74.90,75.61)	
25--29	110720	73141 (66.06)	(65.66,66.46)	
30--34	94871	60672 (63.95)	(63.51,64.39)	
35--39	91839	57727 (62.86)	(62.41,63.30)	
40--44	76912	46931 (61.02)	(60.53,61.51)	
45--49	79837	46300 (57.99)	(57.50,58.48)	
Highest education level attended				
No education	153695	115546 (75.18)	(74.89,75.47)	<0.0001
Primary	80716	56210 (69.64)	(69.19,70.09)	
Secondary	342863	238377 (69.53)	(69.30,69.75)	
Higer	103618	62178 (60.01)	(59.55,60.46)	
Respondent currently working				
No	75702	53353 (70.48)	(70.01,70.94)	<0.0001

Yes	25853	16720 (64.67)	(63.80,65.54)	
N.A.	579338	402237 (69.43)	(69.26,69.60)	
Current marital status				
Never in union	159298	139491 (87.57)	(87.33,87.79)	<0.0001
Currently in union/living with a man	492756	315065 (63.94)	(63.75,64.13)	
Formerly in union/living with a man	28839	17755 (61.57)	(60.74,62.39)	
Wealth index for urban/rural				
Poorest	128793	104190 (80.90)	(80.58,81.21)	<0.0001
Poorer	138134	103372 (74.83)	(74.50,75.16)	
Middle	140354	97773 (69.66)	(69.32,70.00)	
Richer	139494	89453 (64.13)	(63.77,64.48)	
Richest	134117	77521 (57.80)	(57.42,58.18)	
Getting medical help for self				
No problem	291262	189167 (64.95)	(64.69,65.20)	<0.0001
Big problem	159666	118541 (74.24)	(73.95,74.53)	
Not a big problem	229965	164602 (71.58)	(71.32,71.84)	
Covered by health insurance				
No	475922	337435 (70.90)	(70.71,71.09)	<0.0001
Yes	204971	134875 (65.80)	(65.52,66.08)	
Use of internet				
Never	68341	49011 (71.72)	(71.24,72.19)	<0.0001
Yes	33215	21062 (63.41)	(62.63,64.19)	
N.A.	579338	402237 (69.43)	(69.26,69.60)	
**Madagascar**				P value
Age in 5--year groups				
15--19	2106	2042 (96.93)	(95.96,97.68)	<0.0001
20--24	1943	1839 (94.64)	(93.32,95.71)	
25--29	1451	1320 (90.92)	(88.93,92.59)	
30--34	1319	1176 (89.15)	(86.89,91.05)	
35--39	1110	1007 (90.75)	(88.39,92.67)	
40--44	924	828 (89.58)	(86.91,91.76)	
45--49	743	658 (88.56)	(85.53,91.03)	
Highest education level attended				
No education	1545	1515 (98.01)	(97.11,98.64)	<0.0001
Primary	4020	3830 (95.28)	(94.41,96.03)	
Secondary	3622	3259 (89.97)	(88.70,91.11)	
Higer	409	265 (64.85)	(59.13,70.18)	
Respondent currently working				
No	2185	2007 (91.86)	(90.26,93.21)	0.373
Yes	7412	6862 (92.58)	(91.84,93.27)	
N.A.	0	0 (0.00)	--	
Current marital status				
Never in union	2426	2314 (95.38)	(94.32,96.25)	<0.0001
Currently in union/living with a man	5968	5447 (91.27)	(90.35,92.11)	
Formerly in union/living with a man	1203	1108 (92.14)	(90.04,93.83)	
Wealth index for urban/rural				
Poorest	1641	1605 (97.86)	(96.98,98.48)	<0.0001
Poorer	1786	1725 (96.59)	(95.55,97.40)	
Middle	1980	1852 (93.53)	(92.20,94.65)	
Richer	2039	1888 (92.59)	(91.18,93.79)	
Richest	2152	1799 (83.62)	(81.46,85.58)	
Getting medical help for self				
No problem	0	0 (0.00)	--	<0.0001
Big problem	3267	3115 (95.33)	(94.37,96.14)	
Not a big problem	6329	5754 (90.91)	(90.02,91.74)	
Covered by health insurance				
No	9236	8620 (93.33)	(92.68,93.92)	<0.0001
Yes	361	249 (69.14)	(63.09,74.59)	
Use of internet				
Never	8125	7702 (94.80)	(94.18,95.36)	<0.0001
Yes	1472	1167 (79.26)	(76.65,81.65)	
N.A.	0	0 (0.00)	--	
**Mauritania**				P value
Age in 5--year groups				
15--19	1922	1798 (93.50)	(91.91,94.80)	<0.0001
20--24	1365	1178 (86.24)	(83.87,88.32)	

25--29	1344	1071 (79.74)	(76.92,82.29)	
30--34	1069	807 (75.51)	(72.12,78.61)	
35--39	904	625 (69.07)	(65.09,72.79)	
40--44	783	544 (69.43)	(65.37,73.21)	
45--49	570	431 (75.51)	(71.16,79.40)	
Highest education level attended				
No education	2627	2144 (81.60)	(79.76,83.32)	<0.0001
Primary	3099	2560 (82.60)	(80.91,84.17)	
Secondary	2070	1647 (79.54)	(77.17,81.72)	
Higer	162	103 (63.29)	(52.60,72.82)	
Respondent currently working				
No	6389	5263 (82.38)	(81.19,83.50)	<0.0001
Yes	1570	1190 (75.81)	(73.04,78.38)	
N.A.	0	0 (0.00)	--	
Current marital status				
Never in union	2154	2006 (93.13)	(91.57,94.42)	<0.0001
Currently in union/living with a man	4949	3770 (76.18)	(74.68,77.62)	
Formerly in union/living with a man	856	677 (79.10)	(75.52,82.27)	
Wealth index for urban/rural				
Poorest	1484	1290 (86.94)	(84.64,88.94)	<0.0001
Poorer	1534	1294 (84.33)	(81.90,86.50)	
Middle	1568	1301 (82.94)	(80.44,85.18)	
Richer	1606	1256 (78.23)	(75.62,80.64)	
Richest	1766	1312 (74.27)	(71.71,76.68)	
Getting medical help for self				
No problem	0	0 (0.00)	--	<0.0001
Big problem	3341	2806 (84.00)	(82.40,85.47)	
Not a big problem	4618	3647 (78.97)	(77.46,80.40)	
Covered by health insurance				
No	7286	6013 (82.53)	(81.42,83.58)	<0.0001
Yes	673	440 (65.44)	(60.68,69.90)	
Use of internet				
Never	5011	4255 (84.91)	(83.68,86.06)	<0.0001
Yes	2948	2198 (74.57)	(72.48,76.55)	
N.A.	0	0 (0.00)	--	

S4 Table. Subgroup Analysis Among Countries: Unmet needs for Ever Tested for Cervical Cancer

	Sample Size	Unmet Needs (N, %)	95%CI	P value
**Benin**				
Age in 5--year groups				
15--19	1617	1617 (100.00)	100	0.002
20--24	1423	1420 (99.80)	(99.45,99.93)	
25--29	1502	1494 (99.48)	(99.02,99.73)	
30--34	1028	1019 (99.09)	(98.13,99.56)	
35--39	933	923 (98.93)	(97.96,99.44)	
40--44	607	603 (99.28)	(98.19,99.72)	
45--49	595	591 (99.24)	(98.15,99.69)	
Highest education level attended				
No education	4245	4236 (99.80)	(99.62,99.89)	<0.0001
Primary	1547	1542 (99.68)	(99.22,99.87)	
Secondary	1748	1733 (99.16)	(98.62,99.50)	
Higer	166	156 (93.68)	(88.02,96.76)	
Respondent currently working				
No	1872	1865 (99.66)	(99.23,99.85)	0.271
Yes	5834	5802 (99.44)	(99.21,99.61)	
N.A.	0	0 (0.00)	--	
Current marital status				
Never in union	1900	1894 (99.68)	(99.21,99.87)	0.031
Currently in union/living with a man	5409	5382 (99.51)	(99.29,99.66)	
Formerly in union/living with a man	396	391 (98.53)	(96.30,99.42)	
Wealth index for urban/rural				
Poorest	1425	1424 (99.90)	(99.61,99.98)	<0.0001
Poorer	1561	1560 (99.95)	(99.66,99.99)	
Middle	1551	1550 (99.97)	(99.75,100.00)	
Richer	1562	1553 (99.43)	(98.92,99.70)	

Richest	1607	1580 (98.31)	(97.51,98.86)	
Getting medical help for self				
No problem	0	0 (0.00)	--	0.119
Big problem	2404	2397 (99.69)	(99.35,99.85)	
Not a big problem	5302	5270 (99.41)	(99.16,99.59)	
Covered by health insurance				
No	7631	7596 (99.54)	(99.35,99.67)	<0.0001
Yes	74	71 (95.32)	(86.81,98.44)	
Use of internet				
Never	7158	7136 (99.69)	(99.54,99.80)	<0.0001
Yes	548	531 (96.94)	(94.98,98.14)	
N.A.	0	0 (0.00)	--	
**Cameroon**				
Age in 5--year groups				
15--19	3309	3292 (99.47)	(99.13,99.68)	<0.0001
20--24	2417	2374 (98.22)	(97.53,98.72)	
25--29	2426	2344 (96.63)	(95.69,97.38)	
30--34	1972	1885 (95.55)	(94.28,96.55)	
35--39	1498	1413 (94.34)	(92.71,95.62)	
40--44	1091	1014 (93.02)	(91.08,94.57)	
45--49	903	823 (91.11)	(88.70,93.05)	
Highest education level attended				
No education	2778	2758 (99.27)	(98.81,99.55)	<0.0001
Primary	3630	3524 (97.09)	(96.40,97.65)	
Secondary	6158	5912 (96.01)	(95.39,96.55)	
Higer	1049	950 (90.52)	(88.11,92.49)	
Respondent currently working				
No	5190	5073 (97.75)	(97.20,98.19)	<0.0001
Yes	8426	8072 (95.80)	(95.27,96.27)	
N.A.	0	0 (0.00)	--	
Current marital status				
Never in union	4692	4603 (98.11)	(97.63,98.49)	<0.0001
Currently in union/living with a man	7748	7424 (95.82)	(95.25,96.33)	
Formerly in union/living with a man	1175	1117 (95.01)	(93.40,96.25)	
Wealth index for urban/rural				
Poorest	2385	2356 (98.77)	(98.19,99.17)	<0.0001
Poorer	2578	2530 (98.15)	(97.41,98.68)	
Middle	2779	2696 (97.02)	(96.20,97.67)	
Richer	2898	2783 (96.01)	(95.10,96.76)	
Richest	2975	2779 (93.42)	(92.25,94.42)	
Getting medical help for self				
No problem	0	0 (0.00)	--	<0.0001
Big problem	5410	5296 (97.89)	(97.36,98.31)	
Not a big problem	8206	7849 (95.65)	(95.11,96.14)	
Covered by health insurance				
No	13281	12864 (96.85)	(96.49,97.19)	<0.0001
Yes	334	281 (84.05)	(78.59,88.32)	
Use of internet				
Never	9522	9345 (98.15)	(97.81,98.43)	<0.0001
Yes	4094	3799 (92.81)	(91.79,93.70)	
N.A.	0	0 (0.00)	--	
**Gabon**				P value
Age in 5--year groups				
15--19	1127	1098 (97.38)	(94.82,98.69)	<0.0001
20--24	1182	1074 (90.84)	(87.60,93.29)	
25--29	1204	1009 (83.78)	(79.83,87.08)	
30--34	972	775 (79.75)	(75.22,83.64)	
35--39	801	636 (79.50)	(74.52,83.71)	
40--44	738	560 (75.88)	(69.39,81.36)	
45--49	483	361 (74.63)	(67.98,80.30)	
Highest education level attended				
No education	406	363 (89.43)	(82.46,93.83)	<0.0001
Primary	838	762 (90.87)	(88.06,93.08)	
Secondary	4283	3693 (86.23)	(84.34,87.94)	
Higer	980	694 (70.85)	(65.33,75.81)	
Respondent currently working				

No	3678	3254 (88.49)	(86.54,90.18)	<0.0001
Yes	2830	2258 (79.81)	(77.15,82.24)	
N.A.	0	0 (0.00)	--	
Current marital status				
Never in union	2655	2371 (89.30)	(86.96,91.27)	<0.0001
Currently in union/living with a man	3194	2598 (81.35)	(78.93,83.55)	
Formerly in union/living with a man	658	543 (82.52)	(77.27,86.77)	
Wealth index for urban/rural				
Poorest	1144	1030 (90.02)	(87.10,92.34)	<0.0001
Poorer	1321	1166 (88.23)	(85.01,90.84)	
Middle	1357	1170 (86.23)	(82.67,89.15)	
Richer	1425	1185 (83.13)	(79.33,86.35)	
Richest	1260	962 (76.37)	(71.92,80.31)	
Getting medical help for self				
No problem	0	0 (0.00)	--	0.012
Big problem	3291	2851 (86.61)	(84.56,88.43)	
Not a big problem	3216	2662 (82.77)	(80.31,84.98)	
Covered by health insurance				
No	1893	1731 (91.45)	(89.03,93.38)	<0.0001
Yes	4614	3782 (81.95)	(79.95,83.79)	
Use of internet				
Never	2022	1806 (89.34)	(86.90,91.37)	<0.0001
Yes	4485	3706 (82.63)	(80.59,84.49)	
N.A.	0	0 (0.00)	--	
**India**				P value
Age in 5--year groups				
15--19	114575	114261 (99.73)	(99.68,99.76)	<0.0001
20--24	112139	111314 (99.26)	(99.19,99.33)	
25--29	110720	109481 (98.88)	(98.78,98.97)	
30--34	94871	93368 (98.42)	(98.29,98.53)	
35--39	91839	90131 (98.14)	(98.00,98.27)	
40--44	76912	75243 (97.83)	(97.66,97.99)	
45--49	79837	77969 (97.66)	(97.50,97.81)	
Highest education level attended				
No education	153695	151815 (98.78)	(98.70,98.85)	<0.0001
Primary	80716	79406 (98.38)	(98.22,98.52)	
Secondary	342863	338431 (98.71)	(98.65,98.76)	
Higer	103618	102115 (98.55)	(98.42,98.66)	
Respondent currently working				
No	75702	74771 (98.77)	(98.65,98.88)	<0.0001
Yes	25853	25349 (98.05)	(97.79,98.28)	
N.A.	579338	571646 (98.67)	(98.63,98.72)	
Current marital status				
Never in union	159298	158807 (99.69)	(99.65,99.73)	<0.0001
Currently in union/living with a man	492756	484690 (98.36)	(98.31,98.42)	
Formerly in union/living with a man	28839	28269 (98.03)	(97.77,98.25)	
Wealth index for urban/rural				
Poorest	128793	127691 (99.14)	(99.07,99.21)	<0.0001
Poorer	138134	136523 (98.83)	(98.75,98.91)	
Middle	140354	138477 (98.66)	(98.57,98.75)	
Richer	139494	137303 (98.43)	(98.33,98.53)	
Richest	134117	131772 (98.25)	(98.13,98.37)	
Getting medical help for self				
No problem	291262	286995 (98.53)	(98.46,98.60)	<0.0001
Big problem	159666	157832 (98.85)	(98.78,98.92)	
Not a big problem	229965	226940 (98.68)	(98.61,98.75)	
Covered by health insurance				
No	475922	469922 (98.74)	(98.69,98.79)	<0.0001
Yes	204971	201845 (98.48)	(98.39,98.55)	
Use of internet				
Never	68341	67397 (98.62)	(98.49,98.73)	0.237
Yes	33215	32724 (98.52)	(98.31,98.71)	
N.A.	579338	571646 (98.67)	(98.63,98.72)	
**Madagascar**				P value
Age in 5--year groups				
15--19	2106	2104 (99.87)	(99.58,99.96)	<0.0001

20--24	1943	1932 (99.39)	(98.81,99.69)	
25--29	1451	1427 (98.36)	(97.35,98.99)	
30--34	1319	1292 (97.94)	(96.80,98.68)	
35--39	1110	1090 (98.26)	(96.96,99.01)	
40--44	924	899 (97.35)	(95.91,98.29)	
45--49	743	727 (97.92)	(96.61,98.73)	
Highest education level attended				
No education	1545	1539 (99.62)	(99.12,99.84)	<0.0001
Primary	4020	3996 (99.41)	(99.06,99.63)	
Secondary	3622	3556 (98.18)	(97.56,98.64)	
Higer	409	380 (92.85)	(89.64,95.11)	
Respondent currently working				
No	2185	2158 (98.75)	(98.03,99.20)	0.859
Yes	7412	7314 (98.69)	(98.35,98.95)	
N.A.	0	0 (0.00)	--	
Current marital status				
Never in union	2426	2418 (99.67)	(99.34,99.84)	<0.0001
Currently in union/living with a man	5968	5875 (98.44)	(98.02,98.77)	
Formerly in union/living with a man	1203	1179 (98.05)	(96.87,98.79)	
Wealth index for urban/rural				
Poorest	1641	1632 (99.48)	(98.94,99.74)	<0.0001
Poorer	1786	1773 (99.28)	(98.72,99.60)	
Middle	1980	1965 (99.25)	(98.64,99.59)	
Richer	2039	2016 (98.87)	(98.18,99.30)	
Richest	2152	2086 (96.96)	(95.97,97.72)	
Getting medical help for self				
No problem	0	0 (0.00)	--	0.069
Big problem	3267	3236 (99.04)	(98.57,99.35)	
Not a big problem	6329	6236 (98.53)	(98.13,98.84)	
Covered by health insurance				
No	9236	9133 (98.88)	(98.60,99.11)	<0.0001
Yes	361	339 (94.00)	(90.86,96.11)	
Use of internet				
Never	8125	8064 (99.25)	(99.00,99.44)	<0.0001
Yes	1472	1408 (95.66)	(94.24,96.74)	
N.A.	0	0 (0.00)	--	
**Mauritania**				P value
Age in 5--year groups				
15--19	1922	1922 (100.00)	(0.00,0.00)	0.001
20--24	1365	1362 (99.75)	(98.84,99.95)	
25--29	1344	1341 (99.79)	(99.34,99.93)	
30--34	1069	1063 (99.43)	(98.77,99.73)	
35--39	904	898 (99.23)	(97.42,99.77)	
40--44	783	771 (98.41)	(96.72,99.24)	
45--49	570	566 (99.18)	(97.78,99.70)	
Highest education level attended				
No education	2627	2614 (99.49)	(98.86,99.77)	0.162
Primary	3099	3089 (99.66)	(99.29,99.84)	
Secondary	2070	2061 (99.54)	(99.09,99.76)	
Higer	162	159 (98.13)	(92.54,99.55)	
Respondent currently working				
No	6389	6366 (99.65)	(99.41,99.79)	0.038
Yes	1570	1556 (99.12)	(98.16,99.58)	
N.A.	0	0 (0.00)	--	
Current marital status				
Never in union	2154	2154 (100.00)	(0.00,0.00)	0.003
Currently in union/living with a man	4949	4915 (99.32)	(98.93,99.56)	
Formerly in union/living with a man	856	853 (99.69)	(99.22,99.88)	
Wealth index for urban/rural				
Poorest	1484	1480 (99.70)	(98.82,99.92)	0.536
Poorer	1534	1528 (99.60)	(98.46,99.90)	
Middle	1568	1562 (99.63)	(98.93,99.87)	
Richer	1606	1600 (99.62)	(99.13,99.84)	
Richest	1766	1752 (99.21)	(98.58,99.56)	
Getting medical help for self				
No problem	0	0 (0.00)	--	0.815

Big problem	3341	3327 (99.57)	(99.05,99.81)	
Not a big problem	4618	4596 (99.52)	(99.24,99.70)	
Covered by health insurance				
No	7286	7261 (99.66)	(99.42,99.80)	<0.0001
Yes	673	661 (98.25)	(96.71,99.07)	
Use of internet				
Never	5011	4994 (99.67)	(99.34,99.84)	0.090
Yes	2948	2928 (99.32)	(98.88,99.59)	
N.A.	0	0 (0.00)	--	

S5 Table. Subgroup Analysis Among Countries: Unmet needs for NCD needs (i)

	Sample Size	Unmet Needs (N, %)	95%CI	P value
**Benin**				
Age in 5--year groups				
15--19	1617	1617 (100.00)	(0.00,0.00)	<0.0001
20--24	1423	1423 (100.00)	(0.00,0.00)	
25--29	1502	1499 (99.78)	(99.46,99.91)	
30--34	1028	1024 (99.57)	(98.63,99.87)	
35--39	933	927 (99.37)	(98.53,99.74)	
40--44	607	605 (99.60)	(98.60,99.89)	
45--49	595	593 (99.52)	(98.46,99.85)	
Highest education level attended				
No education	4245	4242 (99.94)	(99.82,99.98)	<0. 0001
Primary	1547	1545 (99.87)	(99.56,99.96)	
Secondary	1748	1740 (99.56)	(99.10,99.79)	
Higer	166	160 (95.88)	(90.39,98.29)	
Respondent currently working				
No	1872	1868 (99.82)	(99.35,99.95)	0.572
Yes	5834	5819 (99.73)	(99.56,99.84)	
N.A.	0	0 (0.00)	--	
Current marital status				
Never in union	1900	1896 (99.80)	(99.30,99.94)	0.171
Currently in union/living with a man	5409	5397 (99.78)	(99.61,99.87)	
Formerly in union/living with a man	396	393 (99.24)	(97.36,99.78)	
Wealth index for urban/rural				
Poorest	1425	1424 (99.96)	(99.71,99.99)	<0. 0001
Poorer	1561	1560 (99.95)	(99.66,99.99)	
Middle	1551	1550 (99.97)	(99.75,100.00)	
Richer	1562	1561 (99.93)	(99.54,99.99)	
Richest	1607	1591 (99.01)	(98.32,99.42)	
Getting medical help for self				
No problem	0	0 (0.00)	--	0.752
Big problem	2404	2399 (99.78)	(99.50,99.91)	
Not a big problem	5302	5288 (99.74)	(99.54,99.86)	
Covered by health insurance				
No	7631	7616 (99.80)	(99.66,99.88)	<0. 0001
Yes	74	71 (95.32)	(86.81,98.44)	
Use of internet				
Never	7158	7150 (99.90)	(99.79,99.95)	<0. 0001
Yes	548	537 (97.93)	(96.09,98.91)	
N.A.	0	0 (0.00)	--	
**Cameroon**				
Age in 5--year groups				
15--19	3309	3303 (99.82)	(99.57,99.93)	<0. 0001
20--24	2417	2387 (98.77)	(98.14,99.19)	
25--29	2426	2375 (97.90)	(97.14,98.45)	
30--34	1972	1911 (96.87)	(95.72,97.72)	
35--39	1498	1442 (96.30)	(94.92,97.32)	
40--44	1091	1037 (95.11)	(93.35,96.42)	
45--49	903	840 (93.01)	(90.81,94.72)	
Highest education level attended				
No education	2778	2771 (99.74)	(99.48,99.87)	<0. 0001
Primary	3630	3576 (98.50)	(97.98,98.88)	
Secondary	6158	5983 (97.15)	(96.61,97.61)	
Higer	1049	966 (92.06)	(89.75,93.88)	

Respondent currently working				
No	5190	5106 (98.39)	(97.91,98.76)	<0. 0001
Yes	8426	8189 (97.19)	(96.74,97.58)	
N.A.	0	0 (0.00)	--	
Current marital status				
Never in union	4692	4636 (98.80)	(98.39,99.11)	<0. 0001
Currently in union/living with a man	7748	7519 (97.04)	(96.54,97.46)	
Formerly in union/living with a man	1175	1141 (97.07)	(95.81,97.95)	
Wealth index for urban/rural				
Poorest	2385	2369 (99.34)	(98.90,99.60)	<0. 0001
Poorer	2578	2549 (98.87)	(98.23,99.29)	
Middle	2779	2731 (98.29)	(97.65,98.75)	
Richer	2898	2817 (97.19)	(96.38,97.83)	
Richest	2975	2829 (95.08)	(94.03,95.95)	
Getting medical help for self				
No problem	0	0 (0.00)	--	<0. 0001
Big problem	5410	5341 (98.73)	(98.29,99.06)	
Not a big problem	8206	7954 (96.94)	(96.46,97.35)	
Covered by health insurance				
No	13281	13005 (97.92)	(97.61,98.19)	<0. 0001
Yes	334	291 (86.96)	(81.72,90.87)	
Use of internet				
Never	9522	9422 (98.95)	(98.69,99.16)	<0. 0001
Yes	4094	3873 (94.61)	(93.70,95.39)	
N.A.	0	0 (0.00)	--	
**Gabon**				
Age in 5--year groups				
15--19	1127	1124 (99.74)	(99.04,99.93)	<0.0001
20--24	1182	1150 (97.29)	(95.28,98.46)	
25--29	1204	1123 (93.25)	(90.27,95.36)	
30--34	972	898 (92.35)	(89.14,94.67)	
35--39	801	740 (92.40)	(88.75,94.93)	
40--44	738	649 (89.10)	(82.40,91.88)	
45--49	483	418 (86.47)	(80.66,90.73)	
Highest education level attended				
No education	406	386 (94.94)	(88.53,97.86)	<0.0001
Primary	838	820 (97.85)	(96.76,98.59)	
Secondary	4283	4051 (94.59)	(93.28,95.66)	
Higer	980	844 (86.14)	(81.74,89.62)	
Respondent currently working				
No	3678	3515 (95.58)	(94.26,96.62)	0.003
Yes	2830	2586 (91.39)	(89.42,93.02)	
N.A.	0	0 (0.00)	--	
Current marital status				
Never in union	2655	2543 (95.78)	(94.06,97.02)	0.005
Currently in union/living with a man	3194	2944 (92.16)	(90.38,93.63)	
Formerly in union/living with a man	658	615 (93.41)	(90.24,95.59)	
Wealth index for urban/rural				
Poorest	1144	1112 (97.24)	(95.54,98.30)	<0.0001
Poorer	1321	1274 (96.41)	(94.61,97.62)	
Middle	1357	1277 (94.08)	(91.19,96.06)	
Richer	1425	1332 (93.51)	(90.93,95.39)	
Richest	1260	1106 (87.78)	(84.08,90.71)	
Getting medical help for self				
No problem	0	0 (0.00)	--	0.175
Big problem	3291	3109 (94.45)	(93.03,95.60)	
Not a big problem	3216	2992 (93.05)	(91.26,94.50)	
Covered by health insurance				
No	1893	1813 (95.80)	(93.83,97.16)	0.015
Yes	4614	4288 (92.92)	(91.53,94.10)	
Use of internet				
Never	2022	1941 (96.01)	(94.26,97.24)	0.004
Yes	4485	4160 (92.75)	(91.30,93.97)	
N.A.	0	0 (0.00)	--	
**India**				
Age in 5--year groups				

15--19	114575	114478 (99.92)	(99.89,99.94)	<0.0001
20--24	112139	111697 (99.61)	(99.55,99.66)	
25--29	110720	109906 (99.26)	(99.18,99.34)	
30--34	94871	93888 (98.96)	(98.85,99.06)	
35--39	91839	90672 (98.73)	(98.61,98.84)	
40--44	76912	75759 (98.50)	(98.35,98.64)	
45--49	79837	78475 (98.30)	(98.16,98.43)	
Highest education level attended				
No education	153695	152672 (99.33)	(99.27,99.39)	<0.0001
Primary	80716	79854 (98.93)	(98.80,99.05)	
Secondary	342863	339865 (99.13)	(99.08,99.17)	
Higer	103618	102482 (98.90)	(98.79,99.00)	
Respondent currently working				
No	75702	75124 (99.24)	(99.14,99.32)	<0.0001
Yes	25853	25490 (98.60)	(98.36,98.80)	
N.A.	579338	574260 (99.12)	(99.09,99.16)	
Current marital status				
Never in union	159298	159122 (99.89)	(99.86,99.91)	<0.0001
Currently in union/living with a man	492756	487328 (98.90)	(98.85,98.94)	
Formerly in union/living with a man	28839	28424 (98.56)	(98.33,98.76)	
Wealth index for urban/rural				
Poorest	128793	128183 (99.53)	(99.47,99.58)	<0.0001
Poorer	138134	137136 (99.28)	(99.21,99.34)	
Middle	140354	139167 (99.15)	(99.08,99.22)	
Richer	139494	137973 (98.91)	(98.82,98.99)	
Richest	134117	132414 (98.73)	(98.62,98.83)	
Getting medical help for self				
No problem	291262	288290 (98.98)	(98.92,99.04)	<0.0001
Big problem	159666	158572 (99.32)	(99.25,99.37)	
Not a big problem	229965	228011 (99.15)	(99.09,99.21)	
Covered by health insurance				
No	475922	472072 (99.19)	(99.15,99.23)	<0.0001
Yes	204971	202801 (98.94)	(98.87,99.01)	
Use of internet				
Never	68341	67758 (99.15)	(99.05,99.24)	0.018
Yes	33215	32855 (98.92)	(98.73,99.08)	
N.A.	579338	574260 (99.15)	(99.04,99.24)	
**Madagascar**				
Age in 5--year groups				
15--19	2106	2106 (100.00)	(0.00,0.00)	<0. 0001
20--24	1943	1942 (99.91)	(99.64,99.98)	
25--29	1451	1441 (99.30)	(98.66,99.64)	
30--34	1319	1306 (98.98)	(98.98,99.50)	
35--39	1110	1098 (98.89)	(97.61,99.49)	
40--44	924	909 (98.34)	(97.11,99.05)	
45--49	743	731 (98.42)	(97.15,99.13)	
Highest education level attended				
No education	1545	1543 (99.84)	(99.50,99.95)	<0. 0001
Primary	4020	4011 (99.78)	(99.49,99.90)	
Secondary	3622	3588 (99.06)	(98.54,99.39)	
Higer	409	390 (95.26)	(92.82,96.90)	
Respondent currently working				
No	2185	2172 (99.39)	(98.87,99.67)	0.720
Yes	7412	7360 (99.31)	(99.04,99.50)	
N.A.	0	0 (0.00)	--	
Current marital status				
Never in union	2426	2421 (99.80)	(99.49,99.92)	0.016
Currently in union/living with a man	5968	5918 (99.16)	(98.83,99.40)	
Formerly in union/living with a man	1203	1193 (99.19)	(98.26,99.63)	
Wealth index for urban/rural				
Poorest	1641	1639 (99.89)	(99.54,99.97)	<0. 0001
Poorer	1786	1784 (99.92)	(99.68,99.98)	
Middle	1980	1973 (99.69)	(99.37,99.84)	
Richer	2039	2023 (99.20)	(98.57,99.55)	
Richest	2152	2113 (98.20)	(97.34,98.78)	
Getting medical help for self				

No problem	0	0 (0.00)	--	0.015
Big problem	3267	3255 (99.62)	(99.34,99.78)	
Not a big problem	6329	6277 (99.17)	(98.85,99.41)	
Covered by health insurance				
No	9236	9188 (99.48)	(99.26,99.63)	<0. 0001
Yes	361	344 (95.33)	(92.36,97.18)	
Use of internet				
Never	8125	8100 (99.69)	(99.51,99.81)	<0. 0001
Yes	1472	1432 (97.29)	(96.10,98.12)	
N.A.	0	0 (0.00)	--	
**Mauritania**				
Age in 5--year groups				
15--19	1922	1922 (100.00)	--	0.008
20--24	1365	1362 (99.75)	(98.84,99.95)	
25--29	1344	1343 (99.96)	(99.70,99.99)	
30--34	1069	1065 (99.60)	(98.96,99.85)	
35--39	904	898 (99.27)	(97.40,99.80)	
40--44	783	774 (98.89)	(97.23,99.56)	
45--49	570	568 (99.58)	(98.82,99.85)	
Highest education level attended				
No education	2627	2617 (99.60)	(98.94,99.85)	0.116
Primary	3099	3091 (99.75)	(99.38,99.90)	
Secondary	2070	2065 (99.76)	(99.47,99.89)	
Higer	162	160 (98.38)	(92.23,99.68)	
Respondent currently working				
No	6389	6371 (99.72)	(99.50,99.85)	0.283
Yes	1570	1562 (99.47)	(98.48,99.82)	
N.A.	0	0 (0.00)	--	
Current marital status				
Never in union	2154	2154 (100.00)	--	0.010
Currently in union/living with a man	4949	4924 (99.50)	(99.13,99.71)	
Formerly in union/living with a man	856	855 (99.89)	(99.55,99.97)	
Wealth index for urban/rural				
Poorest	1484	1480 (99.70)	(98.82,99.92)	0.941
Poorer	1534	1529 (99.67)	(98.35,99.93)	
Middle	1568	1563 (99.70)	(98.95,99.91)	
Richer	1606	1602 (99.77)	(99.33,99.92)	
Richest	1766	1758 (99.55)	(99.08,99.78)	
Getting medical help for self				
No problem	0	0 (0.00)	--	0.747
Big problem	3341	3329 (99.64)	(99.10,99.86)	
Not a big problem	4618	4604 (99.70)	(99.46,99.83)	
Covered by health insurance				
No	7286	7268 (99.75)	(99.50,99.87)	0.001
Yes	673	665 (98.85)	(97.61,99.45)	
Use of internet				
Never	5011	4999 (99.78)	(99.42,99.91)	0.145
Yes	2948	2933 (99.50)	(99.10,99.72)	
N.A.	0	0 (0.00)	--	

S6 Table. Subgroup Analysis Among Countries: Unmet needs for NCD needs (ii)

	Sample Size	Unmet Needs (N, %)	95%CI	P value
**Benin**				
Age in 5--year groups				
15--19	1617	1581 (97.74)	(96.75,98.43)	<0.0001
20--24	1423	1313 (92.29)	(90.64,93.68)	
25--29	1502	1379 (91.78)	(90.18,93.14)	
30--34	1028	915 (88.98)	(86.69,90.91)	
35--39	933	837 (89.75)	(87.44,91.68)	
40--44	607	541 (89.15)	(86.22,91.52)	
45--49	595	530 (88.96)	(85.95,91.40)	
Highest education level attended				
No education	4245	3990 (94.00)	(93.17,94.73)	<0.0001
Primary	1547	1417 (91.61)	(89.94,93.03)	
Secondary	1748	1579 (90.33)	(88.77,91.70)	

Higer	166	110 (65.89)	(57.89,73.07)	
Respondent currently working				
No	1872	1766 (94.34)	(93.07,95.39)	<0.0001
Yes	5834	5330 (91.36)	(90.55,92.11)	
N.A.	0	0 (0.00)	--	
Current marital status				
Never in union	1900	1836 (96.60)	(95.57,97.40)	<0.0001
Currently in union/living with a man	5409	4909 (90.75)	(89.89,91.55)	
Formerly in union/living with a man	396	351 (88.53)	(84.46,91.64)	
Wealth index for urban/rural				
Poorest	1425	1361 (95.53)	(94.25,96.54)	<0.0001
Poorer	1561	1475 (94.54)	(93.18,95.64)	
Middle	1551	1455 (93.82)	(92.37,95.01)	
Richer	1562	1430 (91.57)	(89.88,93.00)	
Richest	1607	1374 (85.45)	(83.53,87.18)	
Getting medical help for self				
No problem	0	0 (0.00)	--	0.557
Big problem	2404	2221 (92.37)	(91.12,93.46)	
Not a big problem	5302	4875 (91.95)	(91.13,92.70)	
Covered by health insurance				
No	7631	7045 (92.32)	(91.64,92.94)	<0.0001
Yes	74	51 (68.07)	(56.22,77.97)	
Use of internet				
Never	7158	6668 (93.15)	(92.49,93.76)	<0.0001
Yes	548	428 (78.10)	(74.25,81.52)	
N.A.	0	0 (0.00)	--	
**Cameroon**				
Age in 5--year groups				
15--19	3309	2946 (89.02)	(87.79,90.14)	<0.0001
20--24	2417	1640 (67.84)	(65.67,69.94)	
25--29	2426	1386 (57.12)	(54.76,59.45)	
30--34	1972	1075 (54.48)	(51.81,57.13)	
35--39	1498	789 (52.68)	(49.64,55.69)	
40--44	1091	585 (53.61)	(50.11,57.07)	
45--49	903	481 (53.29)	(49.44,57.10)	
Highest education level attended				
No education	2778	2296 (82.63)	(80.82,84.30)	<0.0001
Primary	3630	2398 (66.07)	(64.30,67.80)	
Secondary	6158	3769 (61.20)	(59.77,62.62)	
Higer	1049	437 (41.70)	(38.14,45.36)	
Respondent currently working				
No	5190	3707 (71.44)	(69.95,72.88)	<0.0001
Yes	8426	5193 (61.64)	(60.40,62.86)	
N.A.	0	0 (0.00)	--	
Current marital status				
Never in union	4692	3591 (76.53)	(75.13,77.88)	<0.0001
Currently in union/living with a man	7748	4605 (59.43)	(58.11,60.74)	
Formerly in union/living with a man	1175	705 (60.00)	(56.71,63.20)	
Wealth index for urban/rural				
Poorest	2385	1866 (78.24)	(76.19,80.16)	<0.0001
Poorer	2578	1873 (72.64)	(70.61,74.59)	
Middle	2779	1753 (63.10)	(60.92,65.23)	
Richer	2898	1766 (60.92)	(58.82,62.97)	
Richest	2975	1643 (55.22)	(53.12,57.30)	
Getting medical help for self				
No problem	0	0 (0.00)	--	<0.0001
Big problem	5410	3915 (72.36)	(70.94,73.74)	
Not a big problem	8206	4986 (60.76)	(59.50,62.01)	
Covered by health insurance				
No	13281	8799 (66.25)	(65.30,67.19)	<0.0001
Yes	334	101 (30.36)	(24.45,36.99)	
Use of internet				
Never	9522	6884 (72.30)	(71.24,73.33)	<0.0001
Yes	4094	2017 (49.26)	(47.43,51.10)	
N.A.	0	0 (0.00)	--	
**Gabon**				

Age in 5--year groups				
15--19	1127	1006 (89.21)	(85.85,91.85)	<0.0001
20--24	1182	898 (75.97)	(71.60,79.85)	
25--29	1204	784 (65.11)	(60.54,69.42)	
30--34	972	583 (59.95)	(54.79,64.91)	
35--39	801	470 (58.76)	(53.08,64.22)	
40--44	738	427 (57.82)	(51.11,64.24)	
45--49	483	257 (53.11)	(46.28,59.82)	
Highest education level attended				
No education	406	307 (75.68)	(67.25,82.50)	<0.0001
Primary	838	627 (74.83)	(70.52,78.71)	
Secondary	4283	3003 (70.10)	(67.80,72.31)	
Higer	980	487 (49.69)	(43.87,55.52)	
Respondent currently working				
No	3678	2738 (74.44)	(72.04,76.71)	<0.0001
Yes	2830	1686 (59.60)	(56.51,62.62)	
N.A.	0	0 (0.00)	--	
Current marital status				
Never in union	2655	2069 (77.94)	(75.12,80.52)	<0.0001
Currently in union/living with a man	3194	1931 (60.44)	(57.58,63.24)	
Formerly in union/living with a man	658	424 (64.47)	(58.53,69.99)	
Wealth index for urban/rural				
Poorest	1144	876 (76.58)	(73.24,79.63)	<0.0001
Poorer	1321	953 (72.14)	(67.86,76.05)	
Middle	1357	927 (68.34)	(63.97,72.40)	
Richer	1425	948 (66.53)	(62.05,70.73)	
Richest	1260	720 (57.11)	(52.31,61.77)	
Getting medical help for self				
No problem	0	0 (0.00)	--	0.702
Big problem	3291	2250 (68.35)	(65.71,70.89)	
Not a big problem	3216	2174 (67.61)	(64.78,70.32)	
Covered by health insurance				
No	1893	1353 (71.46)	(67.84,74.83)	0.024
Yes	4614	3072 (66.56)	(64.27,68.78)	
Use of internet				
Never	2022	1500 (74.20)	(71.05,77.12)	<0.0001
Yes	4485	2924 (65.19)	(62.78,67.53)	
N.A.	0	0 (0.00)	--	
**India**				
Age in 5--year groups				
15--19	114575	105428 (92.02)	(91.79,92.23)	<0.0001
20--24	112139	86363 (77.01)	(76.66,77.36)	
25--29	110720	74755 (67.52)	(67.12,67.91)	
30--34	94871	61877 (65.22)	(64.78,65.66)	
35--39	91839	59090 (64.34)	(63.90,64.78)	
40--44	76912	48051 (62.48)	(61.99,62.96)	
45--49	79837	47559 (59.57)	(59.08,60.06)	
Highest education level attended				
No education	153695	117494 (76.45)	(76.16,76.73)	<0.0001
Primary	80716	57273 (70.96)	(70.51,71.40)	
Secondary	342863	244095 (71.19)	(70.98,71.41)	
Higer	103618	64260 (62.02)	(61.56,62.47)	
Respondent currently working				
No	75702	54540 (72.05)	(71.59,72.50)	<0.0001
Yes	25853	17086 (66.09)	(65.22,66.95)	
N.A.	579338	411496 (71.03)	(70.86,71.20)	
Current marital status				
Never in union	159298	142983 (89.76)	(89.54,89.97)	<0.0001
Currently in union/living with a man	492756	321971 (65.34)	(65.15,65.53)	
Formerly in union/living with a man	28839	18168 (63.00)	(62.17,63.82)	
Wealth index for urban/rural				
Poorest	128793	105962 (82.27)	(81.97,82.57)	<0.0001
Poorer	138134	105467 (76.35)	(76.02,76.67)	
Middle	140354	99944 (71.21)	(70.87,71.55)	
Richer	139494	91957 (65.92)	(65.57,66.27)	
Richest	134117	79792 (59.49)	(59.12,59.87)	

Getting medical help for self				
No problem	291262	193900 (66.57)	(66.32,66.83)	<0.0001
Big problem	159666	121098 (75.84)	(75.56,76.13)	
Not a big problem	229965	168125 (73.11)	(72.85,73.36)	
Covered by health insurance				
No	475922	345099 (72.51)	(72.33,72.70)	<0.0001
Yes	204971	138023 (67.34)	(67.06,67.61)	
Use of internet				
Never	68341	49989 (73.15)	(72.67,73.62)	<0.0001
Yes	33215	21637 (65.14)	(64.36,65.91)	
N.A.	579338	411496 (71.03)	(70.86,71.20)	
**Madagascar**				
Age in 5--year groups				
15--19	2106	2051 (97.38)	(96.46,98.07)	<0.0001
20--24	1943	1837 (94.54)	(93.20,95.63)	
25--29	1451	1321 (91.01)	(89.03,92.66)	
30--34	1319	1174 (89.01)	(86.78,90.90)	
35--39	1110	1007 (90.72)	(88.38,92.63)	
40--44	924	826 (89.41)	(86.76,91.58)	
45--49	743	661 (89.00)	(86.04,91.40)	
Highest education level attended				
No education	1545	1519 (98.29)	(97.46,98.85)	<0.0001
Primary	4020	3838 (95.46)	(94.61,96.19)	
Secondary	3622	3255 (89.87)	(88.61,91.01)	
Higer	409	265 (64.90)	(59.21,70.20)	
Respondent currently working				
No	2185	2024 (92.61)	(91.09,93.89)	0.860
Yes	7412	6854 (92.47)	(91.72,93.16)	
N.A.	0	0 (0.00)	--	
Current marital status				
Never in union	2426	2324 (95.80)	(94.77,96.64)	<0.0001
Currently in union/living with a man	5968	5450 (91.31)	(90.41,92.14)	
Formerly in union/living with a man	1203	1104 (91.76)	(89.62,93.49)	
Wealth index for urban/rural				
Poorest	1641	1606 (97.91)	(97.07,98.52)	<0.0001
Poorer	1786	1726 (96.63)	(95.57,97.44)	
Middle	1980	1857 (93.81)	(92.51,94.90)	
Richer	2039	1889 (92.65)	(91.25,93.83)	
Richest	2152	1799 (83.62)	(81.48,85.56)	
Getting medical help for self				
No problem	0	0 (0.00)	--	<0.0001
Big problem	3267	3111 (95.20)	(94.22,96.02)	
Not a big problem	6329	5767 (91.11)	(90.23,91.92)	
Covered by health insurance				
No	9236	8627 (93.40)	(92.76,93.99)	<0.0001
Yes	361	251 (69.55)	(63.55,74.94)	
Use of internet				
Never	8125	7712 (94.92)	(94.31,95.47)	<0.0001
Yes	1472	1165 (79.17)	(76.57,81.56)	
N.A.				
**Mauritania**				
Age in 5--year groups				
15--19	1922	1823 (94.82)	(93.32,96.00)	<0.0001
20--24	1365	1201 (87.96)	(85.72,89.90)	
25--29	1344	1083 (80.61)	(77.84,83.12)	
30--34	1069	827 (77.38)	(74.09,80.37)	
35--39	904	642 (71.03)	(67.07,74.68)	
40--44	783	567 (72.43)	(68.48,76.06)	
45--49	570	447 (78.35)	(74.26,81.95)	
Highest education level attended				
No education	2627	2177 (82.85)	(81.06,84.50)	<0.0001
Primary	3099	2619 (84.51)	(82.89,86.00)	
Secondary	2070	1692 (81.71)	(79.43,83.80)	
Higer	162	104 (63.93)	(53.22,73.42)	
Respondent currently working				
No	6389	5374 (84.11)	(82.97,85.19)	<0.0001

Yes	1570	1217 (77.55)	(74.85,80.03)	
N.A.	0	0 (0.00)	--	
Current marital status				
Never in union	2154	2039 (94.65)	(93.19,95.80)	<0.0001
Currently in union/living with a man	4949	3858 (77.95)	(76.49,79.35)	
Formerly in union/living with a man	856	694 (81.16)	(81.16,84.18)	
Wealth index for urban/rural				
Poorest	1484	1310 (88.29)	(86.09,90.17)	<0.0001
Poorer	1534	1315 (85.71)	(83.34,87.79)	
Middle	1568	1330 (84.84)	(82.47,86.95)	
Richer	1606	1292 (80.47)	(77.93,82.77)	
Richest	1766	1343 (76.04)	(73.53,78.38)	
Getting medical help for self				
No problem	0	0 (0.00)	--	<0.0001
Big problem	3341	2869 (85.89)	(84.37,87.28)	
Not a big problem	4618	3722 (80.59)	(79.13,81.98)	
Covered by health insurance				
No	7286	6131 (84.15)	(83.08,85.16)	<0.0001
Yes	673	460 (68.42)	(63.74,72.76)	
Use of internet				
Never	5011	4333 (86.48)	(85.31,87.57)	<0.0001
Yes	2948	2258 (76.59)	(74.55,78.51)	
N.A.	0	0 (0.00)	--	

S7 Table. Subgroup Analysis Among Countries: Unmet needs for Contraception

	Sample Size	Unmet Needs (N, %)	95%CI	P value
**Benin**				
Age in 5--year groups				
15--19	1617	246 (15.20)	(13.44,17.14)	<0.0001
20--24	1423	424 (29.81)	(27.33,32.41)	
25--29	1502	461 (30.68)	(28.22,33.26)	
30--34	1028	331 (32.23)	(29.22,35.40)	
35--39	933	299 (32.02)	(28.91,35.29)	
40--44	607	173 (28.51)	(24.80,32.53)	
45--49	595	97 (16.31)	(13.40,19.70)	
Highest education level attended				
No education	4245	1218 (28.69)	(27.25,30.17)	<0.0001
Primary	1547	403 (26.05)	(23.75,28.49)	
Secondary	1748	378 (21.61)	(19.63,23.73)	
Higer	166	33 (19.62)	(14.03,26.74)	
Respondent currently working				
No	1872	427 (22.80)	(20.84,24.89)	<0.0001
Yes	5834	1604 (27.50)	(26.29,28.74)	
N.A.	0	0 (0.00)	--	
Current marital status				
Never in union	1900	232 (12.21)	(10.75,13.84)	<0.0001
Currently in union/living with a man	5409	1754 (32.42)	(31.10,33.77)	
Formerly in union/living with a man	396	45 (11.42)	(8.38,15.37)	
Wealth index for urban/rural				
Poorest	1425	387 (27.13)	(24.76,29.63)	0.191
Poorer	1561	403 (25.84)	(23.58,28.25)	
Middle	1551	443 (28.57)	(26.20,31.06)	
Richer	1562	398 (25.51)	(23.23,27.93)	
Richest	1607	400 (24.86)	(22.69,27.17)	
Getting medical help for self				
No problem	0	0 (0.00)	--	0.020
Big problem	2404	678 (28.21)	(26.32,30.19)	
Not a big problem	5302	1353 (25.51)	(24.28,26.79)	
Covered by health insurance				
No	7631	2014 (26.38)	(25.34,27.46)	0.572
Yes	74	17 (23.47)	(15.06,34.68)	
Use of internet				
Never	7158	1915 (26.75)	(25.67,27.87)	0.006
Yes	548	116 (21.17)	(17.83,24.96)	
N.A.	0	0 (0.00)	--	

**Cameroon**				
Age in 5--year groups				
15--19	3309	369 (11.15)	(9.96,12.46)	<0.0001
20--24	2417	443 (18.33)	(16.64,20.15)	
25--29	2426	487 (20.09)	(18.23,22.08)	
30--34	1972	400 (20.29)	(18.25,22.49)	
35--39	1498	358 (23.88)	(21.34,26.62)	
40--44	1091	230 (21.11)	(18.38,24.12)	
45--49	903	116 (12.85)	(10.51,15.63)	
Highest education level attended				
No education	2778	545 (19.62)	(17.83,21.54)	0.001
Primary	3630	687 (18.93)	(17.52,20.43)	
Secondary	6158	1024 (16.62)	(15.57,17.73)	
Higer	1049	147 (14.05)	(11.59,16.93)	
Respondent currently working				
No	5190	845 (16.28)	(15.15,17.47)	0.005
Yes	8426	1559 (18.50)	(17.52,19.51)	
N.A.	0	0 (0.00)	--	
Current marital status				
Never in union	4692	476 (10.14)	(9.19,11.18)	<0.0001
Currently in union/living with a man	7748	1785 (23.04)	(21.94,24.18)	
Formerly in union/living with a man	1175	142 (12.09)	(10.12,14.39)	
Wealth index for urban/rural				
Poorest	2385	445 (18.67)	(16.82,20.68)	0.001
Poorer	2578	482 (18.71)	(16.95,20.59)	
Middle	2779	540 (19.44)	(17.78,21.23)	
Richer	2898	498 (17.17)	(15.62,18.84)	
Richest	2975	438 (14.71)	(13.32,16.22)	
Getting medical help for self				
No problem	0	0 (0.00)	--	0.219
Big problem	5410	987 (18.24)	(17.05,19.49)	
Not a big problem	8206	1417 (17.26)	(16.32,18.25)	
Covered by health insurance				
No	13281	2353 (17.71)	(16.96,18.49)	0.404
Yes	334	51 (15.12)	(10.26,21.74)	
Use of internet				
Never	9522	1796 (18.86)	(17.95,19.80)	<0.0001
Yes	4094	608 (14.84)	(13.59,16.19)	
N.A.	0	0 (0.00)	--	
**Gabon**				
Age in 5--year groups				
15--19	1127	156 (13.87)	(11.18,17.10)	<0.0001
20--24	1182	320 (27.06)	(23.15,31.35)	
25--29	1204	413 (34.29)	(29.96,38.89)	
30--34	972	305 (31.42)	(26.99,36.21)	
35--39	801	216 (27.03)	(22.56,32.01)	
40--44	738	164 (22.17)	(17.37,27.84)	
45--49	483	67 (13.92)	(10.19,18.74)	
Highest education level attended				
No education	406	135 (33.27)	(25.99,41.44)	0.001
Primary	838	253 (30.18)	(25.96,34.77)	
Secondary	4283	1063 (24.82)	(22.84,26.92)	
Higer	980	190 (19.43)	(15.35,24.28)	
Respondent currently working				
No	3678	909 (24.72)	(22.62,26.93)	0.501
Yes	2830	733 (25.89)	(23.30,28.67)	
N.A.	0	0 (0.00)	--	
Current marital status				
Never in union	2655	442 (16.63)	(14.54,18.95)	<0.0001
Currently in union/living with a man	3194	1074 (33.64)	(31.04,36.34)	
Formerly in union/living with a man	658	126 (19.09)	(14.64,24.50)	
Wealth index for urban/rural				
Poorest	1144	351 (30.70)	(27.51,34.09)	0.002
Poorer	1321	352 (26.66)	(22.89,30.81)	
Middle	1357	330 (24.33)	(20.72,28.34)	
Richer	1425	366 (25.66)	(21.98,29.71)	

Richest	1260	242 (19.24)	(15.84,23.16)	
Getting medical help for self				
No problem	0	0 (0.00)	--	0.110
Big problem	3291	875 (26.59)	(24.29,29.03)	
Not a big problem	3216	766 (23.83)	(21.51,26.32)	
Covered by health insurance				
No	1893	499 (26.35)	(23.21,29.75)	0.413
Yes	4614	1143 (24.77)	(22.86,26.78)	
Use of internet				
Never	2022	577 (28.55)	(25.67,31.61)	0.008
Yes	4485	1064 (23.73)	(21.75,25.84)	
N.A.	0	0 (0.00)	--	
**India**				
Age in 5--year groups				
15--19	114575	2615 (2.28)	(2.17,2.40)	<0.0001
20--24	112139	11692 (10.43)	(10.18,10.68)	
25--29	110720	12728 (11.50)	(11.23,11.76)	
30--34	94871	7908 (8.34)	(8.09,8.59)	
35--39	91839	5414 (5.90)	(5.68,6.11)	
40--44	76912	3456 (4.49)	(4.30,4.69)	
45--49	79837	2324 (2.91)	(2.76,3.07)	
Highest education level attended				
No education	153695	9840 (6.40)	(6.25,6.56)	<0.0001
Primary	80716	5387 (6.67)	(6.45,6.91)	
Secondary	342863	23170 (6.76)	(6.64,6.88)	
Higer	103618	7739 (7.47)	(7.22,7.73)	
Respondent currently working				
No	75702	5466 (7.22)	(6.98,7.47)	<0.0001
Yes	25853	1208 (4.67)	(4.34,5.03)	
N.A.	579338	39463 (6.81)	(6.72,6.90)	
Current marital status				
Never in union	159298	13 (0.01)	(0.00,0.01)	<0.0001
Currently in union/living with a man	492756	46077 (9.35)	(9.24,9.47)	
Formerly in union/living with a man	28839	46 (0.16)	(0.11,0.23)	
Wealth index for urban/rural				
Poorest	128793	9890 (7.68)	(7.49,7.87)	<0.0001
Poorer	138134	9563 (6.92)	(6.74,7.11)	
Middle	140354	9234 (6.58)	(6.40,6.76)	
Richer	139494	8777 (6.29)	(6.12,6.47)	
Richest	134117	8672 (6.47)	(6.26,6.67)	
Getting medical help for self				
No problem	291262	18852 (6.47)	(6.34,6.60)	<0.0001
Big problem	159666	11587 (7.26)	(7.08,7.44)	
Not a big problem	229965	15697 (6.83)	(6.69,6.97)	
Covered by health insurance				
No	475922	35563 (7.47)	(7.37,7.58)	<0.0001
Yes	204971	10574 (5.16)	(5.04,5.28)	
Use of internet				
Never	68341	4487 (6.57)	(6.33,6.81)	0.120
Yes	33215	2187 (6.58)	(6.21,6.97)	
N.A.	579338	39463 (6.81)	(6.72,6.90)	
**Madagascar**				
Age in 5--year groups				
15--19	2106	205 (9.72)	(8.37,11.26)	<0.0001
20--24	1943	203 (10.43)	(8.98,12.09)	
25--29	1451	185 (12.72)	(10.87,14.84)	
30--34	1319	182 (13.79)	(11.81,16.05)	
35--39	1110	185 (16.71)	(14.34,19.40)	
40--44	924	141 (15.27)	(12.85,18.04)	
45--49	743	99 (13.29)	(10.79,16.26)	
Highest education level attended				
No education	1545	278 (17.98)	(16.00,20.14)	<0.0001
Primary	4020	503 (12.52)	(11.39,13.75)	
Secondary	3622	370 (10.21)	(9.12,11.41)	
Higer	409	48 (11.83)	(8.70,15.89)	
Respondent currently working				

No	2185	243 (11.11)	(9.67,12.73)	0.052
Yes	7412	957 (12.91)	(12.07,13.80)	
N.A.	0	0 (0.00)	--	
Current marital status				
Never in union	2426	201 (8.28)	(7.14,9.59)	<0.0001
Currently in union/living with a man	5968	902 (15.11)	(14.09,16.18)	
Formerly in union/living with a man	1203	97 (8.05)	(6.52,9.91)	
Wealth index for urban/rural				
Poorest	1641	292 (17.81)	(15.89,19.92)	<0.0001
Poorer	1786	244 (13.69)	(12.04,15.52)	
Middle	1980	227 (11.46)	(9.94,13.17)	
Richer	2039	226 (11.11)	(9.60,12.81)	
Richest	2152	209 (9.73)	(8.33,11.35)	
Getting medical help for self				
No problem	0	0 (0.00)	--	<0.0001
Big problem	3267	477 (14.59)	(13.30,15.98)	
Not a big problem	6329	723 (11.42)	(10.54,12.36)	
Covered by health insurance				
No	9236	1163 (12.59)	(11.84,13.38)	0.212
Yes	361	37 (10.15)	(7.19,14.15)	
Use of internet				
Never	8125	1044 (12.86)	(12.04,13.71)	0.023
Yes	1472	155 (10.53)	(8.94,12.35)	
N.A.	0	0 (0.00)	--	
**Mauritania**				
Age in 5--year groups				
15--19	1922	168 (8.74)	(7.24,10.52)	0.000
20--24	1365	253 (18.49)	(16.13,21.11)	
25--29	1344	341 (25.36)	(22.54,28.40)	
30--34	1069	292 (27.35)	(24.19,30.77)	
35--39	904	290 (32.08)	(28.40,36.01)	
40--44	783	181 (23.17)	(19.76,26.97)	
45--49	570	105 (18.49)	(14.71,22.97)	
Highest education level attended				
No education	2627	679 (25.83)	(23.78,27.98)	0.000
Primary	3099	673 (21.72)	(19.99,23.55)	
Secondary	2070	259 (12.53)	(10.85,14.42)	
Higer	162	20 (12.32)	(6.84,21.18)	
Respondent currently working				
No	6389	1334 (20.89)	(19.67,22.15)	0.161
Yes	1570	297 (18.89)	(16.57,21.45)	
N.A.	0	0 (0.00)	--	
Current marital status				
Never in union	2154	8 (0.38)	(0.17,0.86)	0.000
Currently in union/living with a man	4949	1578 (31.88)	(30.29,33.51)	
Formerly in union/living with a man	856	45 (5.27)	(3.51,7.84)	
Wealth index for urban/rural				
Poorest	1484	360 (24.29)	(21.80,26.96)	0.000
Poorer	1534	329 (21.47)	(18.98,24.19)	
Middle	1568	355 (22.63)	(19.98,25.51)	
Richer	1606	335 (20.84)	(18.42,23.49)	
Richest	1766	252 (14.24)	(12.51,16.17)	
Getting medical help for self				
No problem	0	0 (0.00)	--	
Big problem	3341	730 (21.86)	(20.16,23.66)	0.039
Not a big problem	4618	901 (19.50)	(18.12,20.96)	
Covered by health insurance				
No	7286	1512 (20.75)	(19.62,21.93)	0.140
Yes	673	119 (17.69)	(14.30,21.68)	
Use of internet				
Never	5011	1150 (22.96)	(21.57,24.41)	0.000
Yes	2948	480 (16.30)	(14.63,18.12)	
N.A.	0	0 (0.00)	--	

S8 Table. Subgroup Analysis Among Countries: Unmet needs for Antenatal Care

	Sample Size	Unmet Needs (N, %)	95%CI	P value
**Benin**				
Age in 5--year groups				
15--19	1617	168 (10.36)	(8.89,12.05)	<0.0001
20--24	1423	604 (42.48)	(39.77,45.24)	
25--29	1502	749 (49.89)	(47.16,52.62)	
30--34	1028	538 (52.31)	(49.01,55.58)	
35--39	933	401 (43.01)	(39.66,46.43)	
40--44	607	187 (30.74)	(26.93,34.83)	
45--49	595	90 (15.20)	(12.37,18.54)	
Highest education level attended				
No education	4245	1952 (45.99)	(44.39,47.60)	<0.0001
Primary	1547	452 (29.22)	(26.81,31.76)	
Secondary	1748	322 (18.45)	(16.60,20.45)	
Higer	166	11 (6.45)	(3.32,12.16)	
Respondent currently working				
No	1872	586 (31.33)	(29.12,33.62)	<0.0001
Yes	5834	2151 (36.87)	(35.55,38.21)	
N.A.	0	0 (0.00)	--	
Current marital status				
Never in union	1900	92 (4.86)	(3.92,6.01)	<0.0001
Currently in union/living with a man	5409	2560 (47.32)	(45.90,48.74)	
Formerly in union/living with a man	396	86 (21.59)	(17.43,26.41)	
Wealth index for urban/rural				
Poorest	1425	788 (55.27)	(52.55,57.96)	<0.0001
Poorer	1561	624 (40.00)	(37.43,42.64)	
Middle	1551	553 (35.64)	(33.11,38.26)	
Richer	1562	449 (28.74)	(26.36,31.24)	
Richest	1607	324 (20.15)	(18.12,22.35)	
Getting medical help for self				
No problem	0	0 (0.00)	--	<0.0001
Big problem	2404	1072 (44.60)	(42.48,46.75)	
Not a big problem	5302	1665 (31.41)	(30.09,32.76)	
Covered by health insurance				
No	7631	2730 (35.77)	(34.62,36.93)	<0.0001
Yes	74	8 (10.54)	(5.31,19.83)	
Use of internet				
Never	7158	2681 (37.46)	(36.27,38.67)	<0.0001
Yes	548	56 (10.21)	(7.82,13.23)	
N.A.	0	0 (0.00)	--	
**Cameroon**				
Age in 5--year groups				
15--19	3309	491 (14.85)	(13.52,16.29)	<0.0001
20--24	2417	956 (39.54)	(37.30,41.82)	
25--29	2426	1175 (48.44)	(46.08,50.82)	
30--34	1972	888 (45.04)	(42.39,47.72)	
35--39	1498	558 (37.29)	(34.40,40.26)	
40--44	1091	260 (23.85)	(20.97,27.00)	
45--49	903	75 (8.34)	(6.34,10.90)	
Highest education level attended				
No education	2778	1555 (55.97)	(53.64,58.27)	<0.0001
Primary	3630	1427 (39.31)	(37.51,41.14)	
Secondary	6158	1311 (21.29)	(20.13,22.49)	
Higer	1049	112 (10.64)	(8.59,13.11)	
Respondent currently working				
No	5190	1353 (26.08)	(24.70,27.50)	<0.0001
Yes	8426	3051 (36.21)	(34.99,37.45)	
N.A.	0	0 (0.00)	--	
Current marital status				
Never in union	4692	440 (9.37)	(8.52,10.30)	<0.0001
Currently in union/living with a man	7748	3647 (47.07)	(45.74,48.41)	
Formerly in union/living with a man	1175	318 (27.02)	(24.22,30.02)	
Wealth index for urban/rural				
Poorest	2385	1129 (47.32)	(44.89,49.76)	<0.0001
Poorer	2578	1031 (39.99)	(37.76,42.26)	
Middle	2779	891 (32.06)	(30.02,34.18)	

Richer	2898	799 (27.57)	(25.74,29.48)	
Richest	2975	555 (18.65)	(17.12,20.28)	
Getting medical help for self				
No problem	0	0 (0.00)	--	<0.0001
Big problem	5410	2145 (39.64)	(38.10,41.20)	
Not a big problem	8206	2260 (27.54)	(26.41,28.70)	
Covered by health insurance				
No	13281	4370 (32.90)	(31.96,33.86)	<0.0001
Yes	334	34 (10.30)	(7.16,14.60)	
Use of internet				
Never	9522	3778 (39.68)	(38.52,40.85)	<0.0001
Yes	4094	626 (15.30)	(14.03,16.65)	
N.A.	0	0 (0.00)	--	
**Gabon**				
Age in 5--year groups				
15--19	1127	95 (8.46)	(6.48,10.97)	<0.0001
20--24	1182	272 (22.98)	(19.37,27.03)	
25--29	1204	293 (24.32)	(20.63,28.44)	
30--34	972	221 (22.77)	(18.85,27.23)	
35--39	801	129 (16.06)	(12.85,19.87)	
40--44	738	67 (9.14)	(6.75,12.26)	
45--49	483	21 (4.29)	(2.62,6.92)	
Highest education level attended				
No education	406	73 (18.05)	(12.86,24.75)	<0.0001
Primary	838	221 (26.36)	(22.48,30.64)	
Secondary	4283	720 (16.80)	(15.18,18.56)	
Higer	980	84 (8.58)	(5.75,12.62)	
Respondent currently working				
No	3678	629 (17.09)	(15.35,18.99)	0.726
Yes	2830	469 (16.59)	(14.54,18.86)	
N.A.	0	0 (0.00)	--	
Current marital status				
Never in union	2655	337 (12.68)	(10.86,14.75)	<0.0001
Currently in union/living with a man	3194	661 (20.70)	(18.63,22.93)	
Formerly in union/living with a man	658	100 (15.19)	(11.41,19.95)	
Wealth index for urban/rural				
Poorest	1144	335 (29.31)	(26.17,32.67)	<0.0001
Poorer	1321	247 (18.66)	(15.47,22.34)	
Middle	1357	226 (16.62)	(13.55,20.22)	
Richer	1425	162 (11.35)	(9.04,14.16)	
Richest	1260	129 (10.21)	(7.86,13.17)	
Getting medical help for self				
No problem	0	0 (0.00)	--	0.020
Big problem	3291	609 (18.50)	(16.60,20.58)	
Not a big problem	3216	489 (15.20)	(13.36,17.24)	
Covered by health insurance				
No	1893	326 (17.23)	(14.78,20.00)	0.743
Yes	4614	772 (16.72)	(15.14,18.43)	
Use of internet				
Never	2022	439 (21.71)	(19.20,24.45)	<0.0001
Yes	4485	659 (14.69)	(13.13,16.39)	
N.A.	0	0 (0.00)	--	
**India**				
Age in 5--year groups				
15--19	114575	2784 (2.43)	(2.31,2.56)	<0.0001
20--24	112139	26303 (23.46)	(23.12,23.80)	
25--29	110720	34470 (31.13)	(30.76,31.51)	
30--34	94871	16899 (17.81)	(17.48,18.15)	
35--39	91839	6728 (7.33)	(7.11,7.55)	
40--44	76912	1648 (2.14)	(2.02,2.27)	
45--49	79837	443 (0.56)	(0.49,0.63)	
Highest education level attended				
No education	153695	23749 (15.45)	(15.22,15.68)	<0.0001
Primary	80716	12088 (14.98)	(14.65,15.30)	
Secondary	342863	42437 (12.38)	(12.23,12.53)	
Higer	103618	10999 (10.62)	(10.34,10.90)	

Respondent currently working				
No	75702	11063 (14.61)	(14.28,14.95)	<0.0001
Yes	25853	2386 (9.23)	(8.78,9.70)	
N.A.	579338	75826 (13.09)	(12.97,13.21)	
Current marital status				
Never in union	151	107 (70.88)	(61.28,78.92)	<0.0001
Currently in union/living with a man	164306	88061 (53.60)	(53.26,53.93)	
Formerly in union/living with a man	1915	1106 (57.75)	(54.66,60.79)	
Wealth index for urban/rural				
Poorest	128793	25305 (19.65)	(19.36,19.94)	<0.0001
Poorer	138134	20645 (14.95)	(14.70,15.20)	
Middle	140354	16799 (11.97)	(11.74,12.20)	
Richer	139494	14365 (10.30)	(10.08,10.52)	
Richest	134117	12161 (9.07)	(8.85,9.29)	
Getting medical help for self				
No problem	67578	31272 (46.28)	(45.74,46.82)	<0.0001
Big problem	40779	24981 (61.26)	(60.63,61.89)	
Not a big problem	58015	33021 (56.92)	(56.36,57.47)	
Covered by health insurance				
No	475922	70281 (14.77)	(14.63,14.91)	<0.0001
Yes	204971	18993 (9.27)	(9.11,9.43)	
Use of internet				
Never	68341	9872 (14.45)	(14.11,14.79)	<0.0001
Yes	33215	3576 (10.77)	(10.31,11.24)	
N.A.	579338	75826 (13.09)	(12.97,13.21)	
**Madagascar**				
Age in 5--year groups				
15--19	2106	508 (24.11)	(22.05,26.29)	<0.0001
20--24	1943	1142 (58.74)	(56.19,61.25)	
25--29	1451	888 (61.20)	(58.22,64.09)	
30--34	1319	694 (52.56)	(49.39,55.72)	
35--39	1110	513 (46.26)	(42.85,49.71)	
40--44	924	262 (28.39)	(25.21,31.80)	
45--49	743	97 (13.11)	(10.64,16.05)	
Highest education level attended				
No education	1545	920 (59.53)	(56.78,62.21)	<0.0001
Primary	4020	1847 (45.94)	(44.15,47.74)	
Secondary	3622	1265 (34.91)	(33.08,36.79)	
Higer	409	73 (17.81)	(13.83,22.64)	
Respondent currently working				
No	2185	658 (30.14)	(27.92,32.45)	<0.0001
Yes	7412	3446 (46.49)	(45.17,47.81)	
N.A.	0	0 (0.00)	--	
Current marital status				
Never in union	2426	367 (15.11)	(13.56,16.81)	<0.0001
Currently in union/living with a man	5968	3218 (53.91)	(52.43,55.39)	
Formerly in union/living with a man	1203	520 (43.21)	(40.04,46.44)	
Wealth index for urban/rural				
Poorest	1641	981 (59.77)	(57.17,62.31)	<0.0001
Poorer	1786	901 (50.48)	(47.92,53.03)	
Middle	1980	873 (44.10)	(41.64,46.59)	
Richer	2039	754 (36.98)	(34.52,39.50)	
Richest	2152	595 (27.65)	(25.26,30.18)	
Getting medical help for self				
No problem	0	0 (0.00)	--	<0.0001
Big problem	3267	1607 (49.18)	(47.20,51.16)	
Not a big problem	6329	2497 (39.45)	(38.05,40.87)	
Covered by health insurance				
No	9236	4034 (43.68)	(42.51,44.86)	<0.0001
Yes	361	70 (19.29)	(14.95,24.54)	
Use of internet				
Never	8125	3780 (46.52)	(45.26,47.78)	<0.0001
Yes	1472	324 (22.04)	(19.64,24.66)	
N.A.	0	0 (0.00)	--	
**Mauritania**				
Age in 5--year groups				

15--19	1922	226 (11.73)	(10.03,13.68)	<0.0001
20--24	1365	452 (33.13)	(30.19,36.21)	
25--29	1344	708 (52.66)	(49.34,55.95)	
30--34	1069	577 (53.94)	(50.16,57.67)	
35--39	904	441 (48.71)	(44.68,52.76)	
40--44	783	299 (38.16)	(34.01,42.49)	
45--49	570	137 (24.07)	(19.95,28.73)	
Highest education level attended				
No education	2627	1175 (44.71)	(42.37,47.07)	<0.0001
Primary	3099	1215 (39.19)	(37.12,41.31)	
Secondary	2070	420 (20.27)	(18.13,22.58)	
Higer	162	30 (18.56)	(11.82,27.92)	
Respondent currently working				
No	6389	2285 (35.77)	(34.33,37.24)	0.757
Yes	1570	553 (35.25)	(32.34,38.28)	
N.A.	0	0 (0.00)	--	
Current marital status				
Never in union	2154	16 (0.73)	(0.42,1.29)	<0.0001
Currently in union/living with a man	4949	2581 (52.15)	(50.42,53.88)	
Formerly in union/living with a man	856	242 (28.29)	(24.71,32.16)	
Wealth index for urban/rural				
Poorest	1484	646 (43.52)	(40.53,46.57)	<0.0001
Poorer	1534	621 (40.46)	(37.41,43.57)	
Middle	1568	612 (39.00)	(35.89,42.21)	
Richer	1606	510 (31.78)	(29.00,34.70)	
Richest	1766	450 (25.49)	(23.17,27.95)	
Getting medical help for self				
No problem	0	0 (0.00)	--	<0.0001
Big problem	3341	1313 (39.30)	(37.27,41.36)	
Not a big problem	4618	1526 (33.04)	(31.37,34.76)	
Covered by health insurance				
No	7286	2651 (36.39)	(35.03,37.77)	0.001
Yes	673	188 (27.90)	(23.83,32.37)	
Use of internet				
Never	5011	2101 (41.92)	(40.27,43.60)	<0.0001
Yes	2948	738 (25.04)	(23.09,27.09)	
N.A.	0	0 (0.00)	--	

S9 Table. Subgroup Analysis Among Countries: Unmet needs for Postnatal Care

	Sample Size	Unmet Needs (N, %)	95%CI	P value
**Benin**				
Age in 5--year groups				
15--19	1617	192 (11.88)	(10.30,13.67)	<0.0001
20--24	1423	773 (54.32)	(51.55,57.07)	
25--29	1502	1058 (70.41)	(67.88,72.82)	
30--34	1028	702 (68.33)	(65.22,71.28)	
35--39	933	522 (55.97)	(52.54,59.34)	
40--44	607	224 (36.82)	(32.79,41.03)	
45--49	595	103 (17.34)	(14.33,20.84)	
Highest education level attended				
No education	4245	2320 (54.66)	(54.66,56.26)	<0.0001
Primary	1547	648 (41.86)	(39.20,44.57)	
Secondary	1748	553 (31.66)	(29.37,34.04)	
Higer	166	53 (31.91)	(24.81,39.96)	
Respondent currently working				
No	1872	719 (38.44)	(36.10,40.83)	<0.0001
Yes	5834	2855 (48.93)	(47.55,50.31)	
N.A.	0	0 (0.00)	--	
Current marital status				
Never in union	1900	125 (6.58)	(5.47,7.90)	<0.0001
Currently in union/living with a man	5409	3310 (61.20)	(59.80,62.58)	
Formerly in union/living with a man	396	139 (34.95)	(29.89,40.39)	
Wealth index for urban/rural				
Poorest	1425	835 (58.60)	(55.90,61.25)	<0.0001
Poorer	1561	738 (47.31)	(44.66,49.98)	

Middle	1551	714 (46.03)	(43.34,48.75)	
Richer	1562	680 (43.52)	(40.87,46.20)	
Richest	1607	607 (37.77)	(35.26,40.34)	
Getting medical help for self				
No problem	0	0 (0.00)	--	0.108
Big problem	2404	1150 (47.84)	(45.70,49.99)	
Not a big problem	5302	2424 (45.72)	(44.28,47.16)	
Covered by health insurance				
No	7631	3553 (46.56)	(45.36,47.76)	0.002
Yes	74	21 (28.28)	(18.88,40.04)	
Use of internet				
Never	7158	3418 (47.76)	(46.51,49.00)	<0.0001
Yes	548	156 (28.44)	(24.59,32.62)	
N.A.	0	0 (0.00)	--	
**Cameroon**				
Age in 5--year groups				
15--19	3309.113998	442 (13.35)	(12.09,14.71)	<0.0001
20--24	2417.11381	1008 (41.72)	(39.46,44.01)	
25--29	2425.843327	1291 (53.22)	(50.86,55.57)	
30--34	1972.438635	975 (49.41)	(46.74,52.09)	
35--39	1497.670711	674 (44.98)	(41.98,48.02)	
40--44	1090.58082	277 (25.41)	(22.47,28.60)	
45--49	902.9108506	73 (8.06)	(6.08,10.60)	
Highest education level attended				
No education	2778.43674	1428 (51.38)	(49.04,53.72)	<0.0001
Primary	3630.107295	1389 (38.26)	(36.47,40.08)	
Secondary	6158.167946	1688 (27.41)	(26.14,28.72)	
Higer	1048.960169	235 (22.38)	(19.51,25.54)	
Respondent currently working				
No	5189.643664	1529 (29.47)	(28.03,30.94)	<0.0001
Yes	8426.028486	3210 (38.10)	(36.87,39.34)	
N.A.		0 (0.00)	(0.00,0.00)	
Current marital status				
Never in union	4691.963739	550 (11.71)	(10.75,12.75)	<0.0001
Currently in union/living with a man	7748.262198	3827 (49.39)	(48.05,50.72)	
Formerly in union/living with a man	1175.446213	363 (30.90)	(27.91,34.05)	
Wealth index for urban/rural				
Poorest	2385.263928	1088 (45.61)	(43.19,48.06)	<0.0001
Poorer	2577.845551	1021 (39.59)	(37.38,41.85)	
Middle	2778.831027	976 (35.13)	(33.04,37.28)	
Richer	2898.468403	864 (29.82)	(27.93,31.79)	
Richest	2975.263241	790 (26.56)	(24.77,28.42)	
Getting medical help for self				
No problem	0	0 (0.00)	--	<0.0001
Big problem	5410.090929	2149 (39.73)	(38.19,41.28)	
Not a big problem	8205.581222	2590 (31.56)	(30.39,30.39)	
Covered by health insurance				
No	13281.37325	4686 (35.29)	(34.33,36.25)	<0.0001
Yes	334.2989042	53 (15.81)	(11.84,20.79)	
Use of internet				
Never	9521.786508	3693 (38.79)	(37.64,39.95)	<0.0001
Yes	4093.885642	1046 (25.56)	(24.00,27.17)	
N.A.	0	0 (0.00)	--	
**Gabon**				
Age in 5--year groups				
15--19	1127	66 (5.87)	(4.11,8.32)	<0.0001
20--24	1182	216 (18.27)	(14.85,22.27)	
25--29	1204	251 (20.87)	(17.34,24.89)	
30--34	972	205 (21.09)	(17.20,25.58)	
35--39	801	120 (14.99)	(11.63,19.12)	
40--44	738	88 (11.87)	(8.32,16.68)	
45--49	483	19 (3.89)	(2.08,7.16)	
Highest education level attended				
No education	406	83 (20.50)	(14.18,28.68)	0.001
Primary	838	177 (21.10)	(17.66,25.02)	
Secondary	4283	594 (13.86)	(12.27,15.62)	

Higer	980	111 (11.33)	(8.05,15.71)	
Respondent currently working				
No	3678	566 (15.40)	(13.60,17.39)	0.365
Yes	2830	399 (14.08)	(12.11,16.32)	
N.A.	0	0 (0.00)	(0.00,0.00)	
Current marital status				
Never in union	2655	243 (9.14)	(7.43,11.20)	<0.0001
Currently in union/living with a man	3194	650 (20.34)	(18.18,22.68)	
Formerly in union/living with a man	658	72 (11.01)	(7.84,15.25)	
Wealth index for urban/rural				
Poorest	1144	226 (19.75)	(17.22,22.56)	<0.0001
Poorer	1321	241 (18.27)	(14.92,22.18)	
Middle	1357	193 (14.25)	(11.32,17.79)	
Richer	1425	174 (12.24)	(9.53,15.60)	
Richest	1260	129 (10.28)	(7.72,13.56)	
Getting medical help for self				
No problem	0	0 (0.00)	--	<0.0001
Big problem	3291	588 (17.87)	(15.84,20.09)	
Not a big problem	3216	377 (11.72)	(10.02,13.65)	
Covered by health insurance				
No	1893	262 (13.85)	(11.52,16.57)	0.382
Yes	4614	703 (15.23)	(13.61,17.00)	
Use of internet				
Never	2022	378 (18.69)	(16.23,21.43)	<0.0001
Yes	4485	587 (13.08)	(11.51,14.84)	
N.A.	0	0 (0.00)	--	
**India**				
Age in 5--year groups				
15--19	114575	2921 (2.55)	(2.42,2.69)	<0.0001
20--24	112139	26403 (23.54)	(23.20,23.89)	
25--29	110720	34646 (31.29)	(30.90,31.68)	
30--34	94871	17324 (18.26)	(17.91,18.61)	
35--39	91839	6509 (7.09)	(6.86,7.32)	
40--44	76912	1486 (1.93)	(1.81,2.06)	
45--49	79837	395 (0.49)	(0.43,0.57)	
Highest education level attended				
No education	153695	19361 (12.60)	(12.39,12.81)	<0.0001
Primary	80716	10813 (13.40)	(13.08,13.72)	
Secondary	342863	45207 (13.19)	(13.03,13.35)	
Higer	103618	14302 (13.80)	(13.47,14.14)	
Respondent currently working				
No	75702	11157 (14.74)	(14.39,15.10)	<0.0001
Yes	25853	2250 (8.70)	(8.23,9.20)	
N.A.	579338	76275 (13.17)	(13.04,13.29)	
Current marital status				
Never in union	159298	97 (0.06)	(0.05,0.08)	<0.0001
Currently in union/living with a man	492756	88575 (17.98)	(17.82,18.13)	
Formerly in union/living with a man	28839	1010 (3.50)	(3.21,3.83)	
Wealth index for urban/rural				
Poorest	128793	21318 (16.55)	(16.27,16.84)	<0.0001
Poorer	138134	19162 (13.87)	(13.63,14.12)	
Middle	140354	17311 (12.33)	(12.10,12.57)	
Richer	139494	16510 (11.84)	(11.60,12.08)	
Richest	134117	15382 (11.47)	(11.22,11.73)	
Getting medical help for self				
No problem	291262	35866 (12.31)	(12.14,12.49)	<0.0001
Big problem	159666	22399 (14.03)	(13.80,14.26)	
Not a big problem	229965	31417 (13.66)	(13.47,13.86)	
Covered by health insurance				
No	475922	69155 (14.53)	(14.39,14.68)	<0.0001
Yes	204971	20528 (10.02)	(9.85,10.18)	
Use of internet				
Never	68341	9093 (13.31)	(12.96,13.66)	0.602
Yes	33215	4314 (12.99)	(12.46,13.54)	
N.A.	579338	76275 (13.17)	(13.04,13.29)	
**Madagascar**				

Age in 5--year groups				
15--19	2106	421 (19.96)	(18.04,22.03)	<0.0001
20--24	1943	908 (46.73)	(44.16,49.32)	
25--29	1451	702 (48.36)	(45.36,51.36)	
30--34	1319	571 (43.31)	(40.19,46.48)	
35--39	1110	405 (36.52)	(33.27,39.90)	
40--44	924	193 (20.87)	(18.14,23.90)	
45--49	743	83 (11.22)	(8.94,14.00)	
Highest education level attended				
No education	1545	786 (50.86)	(48.10,53.61)	<0.0001
Primary	4020	1461 (36.34)	(34.62,38.09)	
Secondary	3622	976 (26.94)	(25.26,28.69)	
Higer	409	61 (14.92)	(11.42,19.27)	
Respondent currently working				
No	2185	574 (26.28)	(24.14,28.54)	<0.0001
Yes	7412	2709 (36.55)	(35.29,37.83)	
N.A.	0	0 (0.00)	(0.00,0.00)	
Current marital status				
Never in union	2426	301 (12.41)	(11.00,13.97)	<0.0001
Currently in union/living with a man	5968	2585 (43.31)	(41.85,44.79)	
Formerly in union/living with a man	1203	398 (33.05)	(30.09,36.15)	
Wealth index for urban/rural				
Poorest	1641	809 (49.30)	(46.67,51.93)	<0.0001
Poorer	1786	728 (40.76)	(38.27,43.29)	
Middle	1980	661 (33.37)	(31.05,35.77)	
Richer	2039	608 (29.83)	(27.50,32.26)	
Richest	2152	478 (22.22)	(20.03,24.57)	
Getting medical help for self				
No problem	0	0 (0.00)	--	<0.0001
Big problem	3267	1222 (37.41)	(35.52,39.34)	
Not a big problem	6329	2061 (32.56)	(31.23,33.93)	
Covered by health insurance				
No	9236	3219 (34.86)	(33.74,35.99)	<0.0001
Yes	361	64 (17.76)	(13.30,23.32)	
Use of internet				
Never	8125	3017 (37.13)	(35.92,38.36)	<0.0001
Yes	1472	267 (18.12)	(15.87,20.61)	
N.A.	0	0 (0.00)	--	
**Mauritania**				
Age in 5--year groups				
15--19	1922	229 (11.89)	(10.20,13.82)	<0.0001
20--24	1365	518 (37.91)	(34.82,41.11)	
25--29	1344	777 (57.86)	(54.58,61.07)	
30--34	1069	640 (59.82)	(56.13,63.41)	
35--39	904	494 (54.63)	(50.59,58.61)	
40--44	783	299 (38.19)	(34.09,42.47)	
45--49	570	137 (23.95)	(19.80,28.66)	
Highest education level attended				
No education	2627	1188 (45.22)	(42.87,47.58)	<0.0001
Primary	3099	1302 (42.01)	(39.91,44.14)	
Secondary	2070	556 (26.87)	(24.45,29.44)	
Higer	162	47 (29.01)	(20.27,39.65)	
Respondent currently working				
No	6389	2456 (38.45)	(36.98,39.94)	0.224
Yes	1570	637 (40.55)	(37.52,43.66)	
N.A.	0	0 (0.00)	--	
Current marital status				
Never in union	2154	15 (0.70)	(0.38,1.28)	<0.0001
Currently in union/living with a man	4949	2787 (56.32)	(54.60,58.02)	
Formerly in union/living with a man	856	291 (33.98)	(30.12,38.06)	
Wealth index for urban/rural				
Poorest	1484	664 (44.74)	(41.73,47.79)	<0.0001
Poorer	1534	614 (40.01)	(36.98,43.12)	
Middle	1568	669 (42.64)	(39.45,45.88)	
Richer	1606	597 (37.16)	(34.21,40.22)	
Richest	1766	550 (31.13)	(28.63,33.74)	

Getting medical help for self				
No problem		0 (0.00)	(0.00,0.00)	0.230
Big problem	3341	1330 (39.82)	(37.79,41.90)	
Not a big problem	4618	1763 (38.17)	(36.43,39.94)	
Covered by health insurance				
No	7286	2845 (39.05)	(37.66,40.45)	0.379
Yes	673	248 (36.84)	(32.31,41.62)	
Use of internet				
Never	5011	2128 (42.48)	(40.82,44.15)	<0.0001
Yes	2948	965 (32.72)	(30.55,34.97)	
N.A.	0	0 (0.00)	--	

S2 Fig. Subgroup Analysis of Unmet Needs for Ever Had Blood Pressure Measured Across Countries

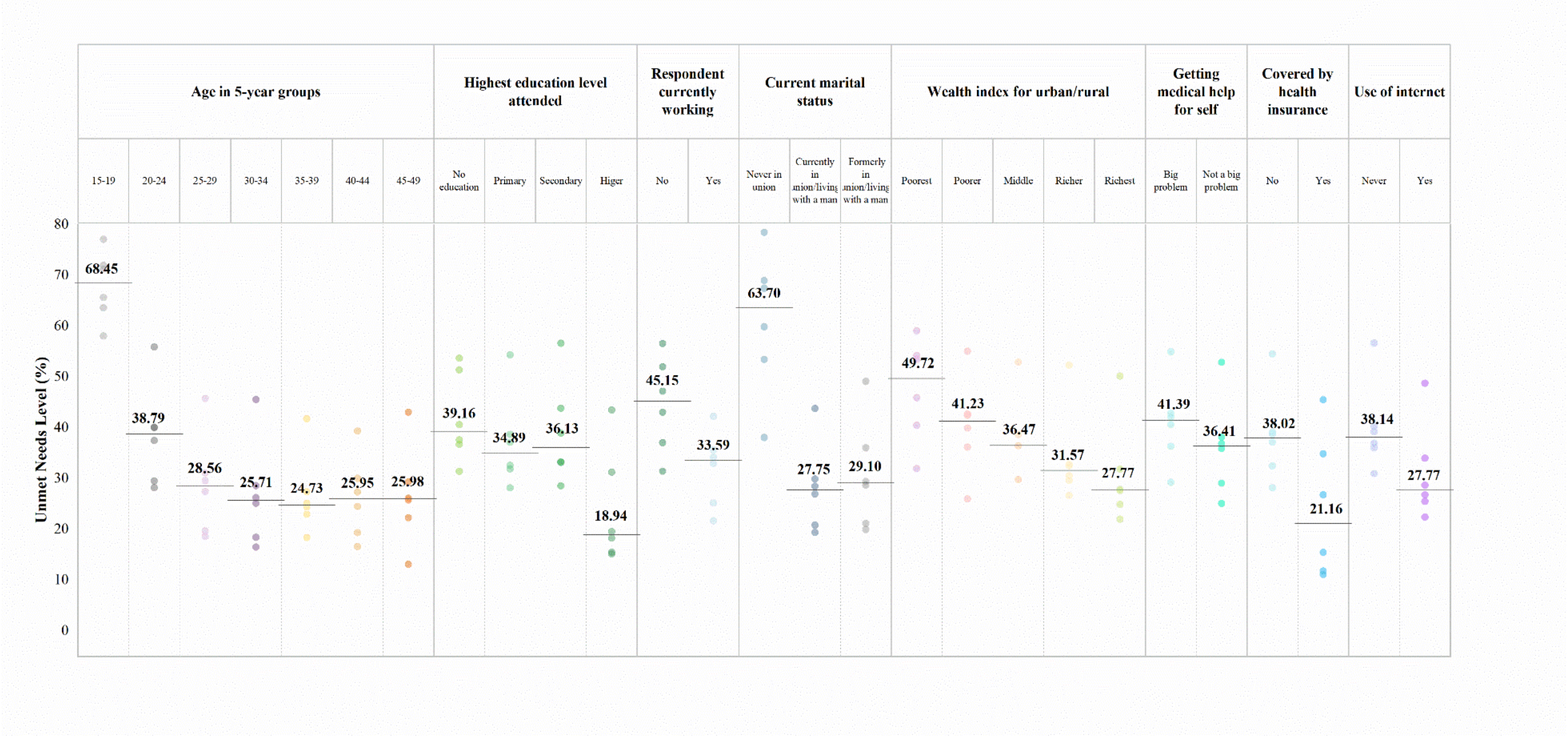

S3 Fig. Subgroup Analysis of Unmet Needs for Ever Had Blood Glucose Measured Across Countries

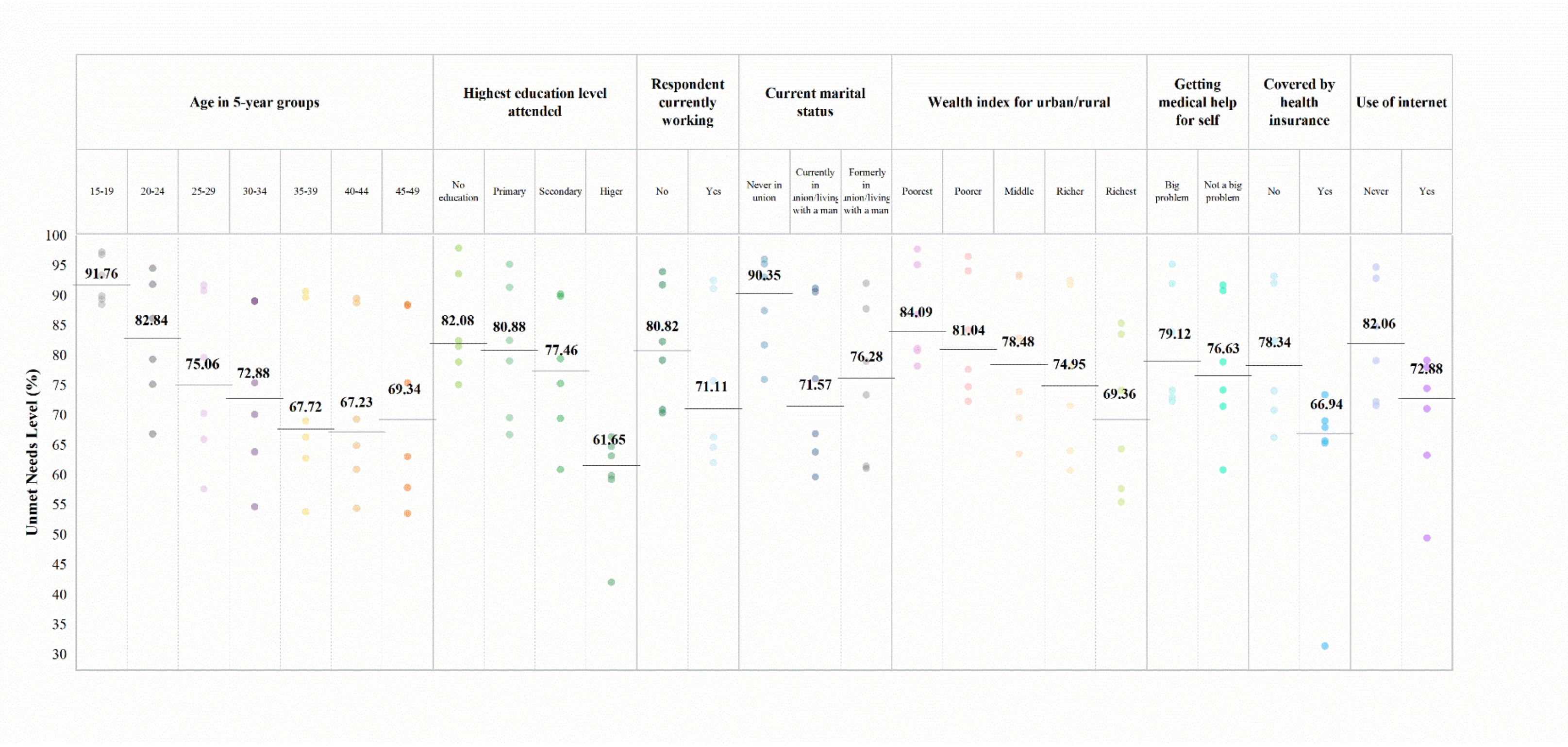

S4 Fig. Subgroup Analysis of Unmet Needs for Ever Tested for Cervical Cancer Across Countries

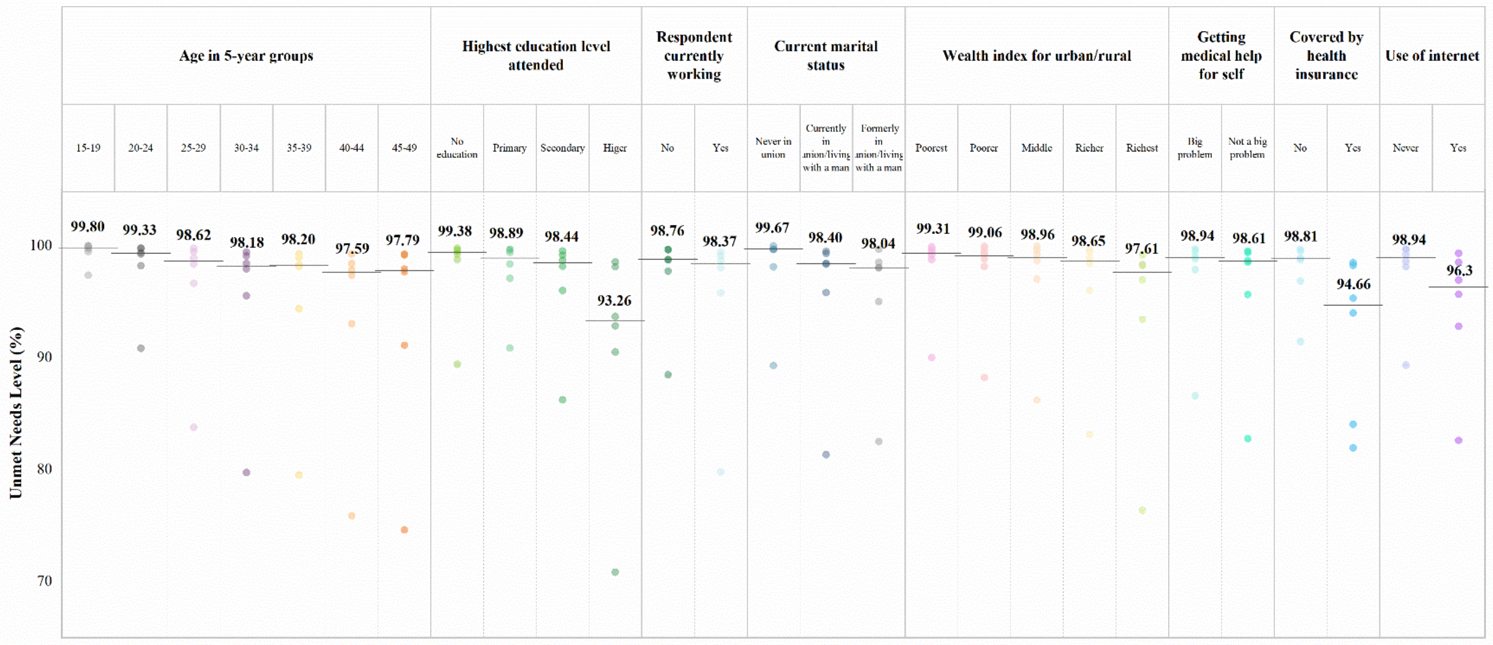
Age in 5-year groups
Highest education level attended
Respondent currently working
Current marital status
Wealth index for urban/rural
Getting medical help for self
Covered by health insurance
Use of internet
15-19
20-24
25-29
30-34
35-39
40-44
45-49
No education
Primary
Secondary
Higer
No
Yes
Never in union
Currently in union/living with a man
Formerly in union/living with a man
Poorest
Poorer
Middle
Richer
Richest
Big problem
Not a big problem
No
Yes
Never
Yes
Unmet Needs Level (%)
100
90
80
70
99.80
99.33
98.62
98.18
98.20
97.59
97.79
99.38
98.89
98.44
93.26
98.76
98.37
99.67
98.40
98.04
99.31
99.06
98.96
98.65
97.61
98.94
98.61
98.81
94.66
98.94
96.3
