# Supplementary material for: Unmet needs for non-communicable diseases and sexual and reproductive health services among women of reproductive age in low-and-middle-income countries: evidence from the Demographic and Health Surveys
Source: BMJ Open. 2026 Feb 9;16(2):e105422. doi: 10.1136/bmjopen-2025-105422 (PMC12887515; doi:10.1136/bmjopen-2025-105422)
Supplement: Supplementary file 2 [file bmjopen-2025-105422.reviewer_comments.pdf]

PEER REVIEW HISTORY

BMJ Open publishes all reviews undertaken for accepted manuscripts. Reviewers are asked to complete a checklist review form and are provided with free text boxes to elaborate on their assessment. These free text comments are reproduced below.

ARTICLE DETAILS

Title (Provisional)

Unmet Needs for Non-Communicable Diseases and Sexual and Reproductive Health Services among women of reproductive age in Low-And-Middle-Income Countries: Evidence from the Demographic and Health Surveys

Authors

Yin, Yishu; Du, Yeting; Zheng, Zhi-Jie; Ren, Minghui; Wang, Minmin; Jin, Yinzi

---

VERSION 1 - REVIEW

**Reviewer**	**1**
**Name**	**Nongkynrih, Baridalyne**
**Affiliation**	**All India Institute of Medical Sciences, Centre for Community Medicine**
**Date**	**25-Jul-2025**
**COI**	**None**

The authors have not explained the criteria or rationale of selecting these 6 countries out of all LMICs

Combination of unmet need NCD for NCD and SRH doesn't have any logical explanation. These are two different areas and both have different trajectories as far as health programs are concerned. In most countries NCD prevention and control is a recent inclusion into national health programs hence the unmet need is expected to be high; whereas sexual and reproductive health programs have been functioning since more than 5 decades and the programs are mature in most countries.

Combining data for all 6 countries together is an over-simplification of complex issues which govern the unmet needs for NCD and SRH

**Reviewer**	**2**
**Name**	**de Carvalho, Angelita Alves**
**Affiliation**	**National School of Statistical Sciences, Program of Population, Territory and Public Policies**

**Date**	**09-Oct-2025**
**COI**	**None**

Introductory Remarks

The manuscript addresses a highly relevant topic that has been extensively studied in the health and demographic literature. Despite this, it innovates by attempting to aggregate a set of unmet needs into a single indicator. Some aspects still require further explanation and exploration. My requests and recommendations are intended to make the article more contributive and reflective of its findings and their limitations. Here are my specific comments:

Major Comments

• Clarity and Presentation: The acronym "NCD" is introduced in the text, but its full description is provided later. It should be defined in the title, abstract, and on its first appearance in the main text. The names of the countries analyzed should also be included in the abstract, and the authors should present percentages with one decimal place for consistency and precision. Additionally, it is important for the authors to justify their choice of countries, as despite being LMICs, these countries have unique characteristics that differentiate them from the vast majority of other LMICs.

• Contextualization and Discussion: The authors must discuss the impact of the COVID-19 pandemic on the indicators analyzed in Madagascar, including a review of studies that have already reported on this topic. Furthermore, the discussion lacks a detailed analysis of each of the estimated indicators (NCD prevention, contraceptive needs, antenatal care, and postnatal care) in the countries analyzed. A more granular discussion is needed, especially since numerous individual estimates of these topics already exist in the literature.

• Data Source: Regarding the data source, it is puzzling that, given the recent events surrounding DHS funding, the authors do not comment—even in a note—on the impacts of the interruption of DHS research and the broader importance of this data source for studies on these topics in developing countries.

• Unmet Contraceptive Needs: The unmet contraceptive needs indicator, although recommended by the WHO, is not the most widely used measure in the fields of public health and demography. I suggest that the authors justify their choice and also discuss the possibility of using other indicators, such as those discussed in the following texts. I believe that presenting the results of these studies and engaging in the methodological discussions would significantly strengthen the manuscript.

o BRADLEY, Sarah E.K.; CASTERLINE, John B. Understanding unmet need: history, theory, and measurement. Studies in family planning, v. 45, n. 2, p. 123-150, 2014.

o Bradley, Sarah E. K., Trevor Croft, Joy Fishel, and Charles Westoff. 2012. Revising Unmet Need for Family Planning. DHS Analytical Studies No. 25. Calverton, MD: ICF International.

o KARRA, Mahesh. Measurement of unmet need for contraception: a counterfactual approach. Studies in Family Planning, v. 53, n. 4, p. 657-680, 2022.

• NCD Indicator Definition: Beyond this indicator, I believe the most complicated aspect is the definition used for the unmet needs indicator in NCD prevention, which includes blood pressure measurement, blood glucose measurement, and cervical cancer screening. I would like the authors to justify the relevance of these indicators, citing relevant studies on the topic. I believe that the issue of glucose and blood pressure testing is more specifically associated with pregnant women, not with all women in the study's age group. Furthermore, the chosen age group does not align with the recommended age for cervical cancer screening (women aged 25 to 64). I ask that the authors justify their decision to restrict the age group to women up to 49 years old. Why was breast cancer screening (recommended for women between 50 and 69 years old), a key preventive health test for women, not included? If a consensus on this indicator does not exist in the literature, it is essential for the authors to present the debate, justify their specific choices, and acknowledge the limitations that these choices entail. In other words, they need to acknowledge that the indicator fails to capture an important dimension of access to reproductive health.

• Interpretation of Findings: Despite being seemingly low, the percentage of women with unmet contraceptive needs cannot be overlooked and must be discussed within the context of reproductive and sexual rights. The data on unmet antenatal and postnatal care services reflect a similar situation, given the extremely low fertility rates and persistently high maternal mortality rates in these countries.

• Figures: Finally, the figures must be redone. They are currently illegible, even when magnified, which compromises the manuscript's presentation and clarity.

VERSION 1 - AUTHOR RESPONSE

Reviewer 1 Comments

1 The authors have not explained the criteria or rationale of selecting these 6 countries out of all LMICs

Authors' Response: We thank the reviewer for this suggestion. We had extended the country selection criteria in the "Methods, data sources" section as " Considering that DHS began to collect information on NCD services in 2015, we adopted the following inclusion criteria: (1) conducted after 2015 and dataset was accessible through the DHS database (2) respond to three NCDs services: "ever had blood pressure measured", "ever had blood sugar measured" and

“tested for cervical cancer”(3) respond to questions like “unmet need for contraception” (4) respond to questions of antenatal care including “prenatal: no one” “timing of 1st antenatal check (months)” “number of antenatal visits during pregnancy” and “place of delivery” (5) consisted questions and valid responses for postnatal care “baby postnatal check within 2 months". Six countries were included in our study: Benin (2017), Cameroon (2018), Gabon (2019), India (2019), Madagascar (2021), and Mauritania (2019).”

2 Combination of unmet need NCD for NCD and SRH doesn’t have any logical explanation. These are two different areas and both have different trajectories as far as health programs are concerned. In most countries NCD prevention and control is a recent inclusion into national health programs hence the unmet need is expected to be high; whereas sexual and reproductive health programs have been functioning since more than 5 decades and the programs are mature in most countries.

- **Authors’ Response：** We appreciate this critical observation and agree that SRH and NCD programs have distinct historical trajectories and maturity levels. Our intention was not to compare them as equivalent entities, but to analyze them jointly from a life-course perspective of women's health. As the disease spectrum evolves, the burden of NCDs among women of reproductive age is increasing. While SRH services are indeed more established, significant coverage gaps persist. Examining both areas together allows for a systematic assessment of women's comprehensive health service needs—spanning both their reproductive years and chronic disease prevention—and helps identify structural weaknesses within the health system. Our findings precisely align with your point regarding program maturity. The data show that unmet needs for SRH services (e.g., contraception, antenatal and postnatal care) are relatively lower, reflecting the progress made by decades of program investment. In stark contrast, the

unmet needs for NCD prevention—particularly cervical cancer screening—are strikingly high. This stark disparity underscores that, despite advancements in SRH, NCD prevention systems have not yet been effectively integrated or scaled to reach target populations in many LMICs. Our study aims to highlight the urgent need to address the growing NCD burden alongside ongoing SRH efforts, a call increasingly echoed by the WHO and other global health bodies advocating for integrated service delivery in primary care.

3 Combining data for all 6 countries together is an over-simplification of complex issues which govern the unmet needs for NCD and SRH

- Authors' Response: We thank the reviewer for this comment. The primary aim of combining data from the six countries was to identify common patterns and social determinants of unmet needs that exist across multiple LMICs, thereby providing a basis for cross-national learning and collaboration. As outlined in Discussion paragraph 7, we identified higher education, better economic status, ready access to healthcare, health-insurance coverage and greater internet use as universal factors that lower unmet needs for both NCD and SRH services across the six countries.

To prevent readers from concluding that "one size fits all", we simultaneously inserted four country-specific segments (paragraphs 3-6) that dissect indicator-by-indicator variation:

1) Paragraph 3 compares contraception, ANC and PNC across all six countries and adds COVID-19-related evidence explaining Madagascar's spike in ANC unmet need.
2) New paragraph 4 details blood-pressure screening performance—Gabon and Cameroon integrate it into routine primary care, whereas Mauritania's equipment shortages drive the highest never-measured rate (53.8 %).
3) New paragraph 5 unpacks blood-glucose testing—Cameroon and India achieve the lowest

gaps (≈ 65-69 %), while Benin and Madagascar exceed 90 %, partly because 2021 pandemic disruptions broke supply chains.

4) Paragraph 6 retains the original country-by-country analysis of cervical-cancer screening, highlighting Gabon's shorter (3-year) interval and the absence of formal guidelines elsewhere

By presenting both the overarching determinants and these detailed national contrasts, we aim to highlight commonalities while remaining mindful of the unique context of each country.

Reviewer 2 Comments

Introductory Remarks

The manuscript addresses a highly relevant topic that has been extensively studied in the health and demographic literature. Despite this, it innovates by attempting to aggregate a set of unmet needs into a single indicator. Some aspects still require further explanation and exploration. My requests and recommendations are intended to make the article more contributive and reflective of its findings and their limitations. Here are my specific comments:

1 Clarity and Presentation: The acronym "NCD" is introduced in the text, but its full description is provided later. It should be defined in the title, abstract, and on its first appearance in the main text. The names of the countries analyzed should also be included in the abstract, and the authors should present percentages with one decimal place for consistency and precision. Additionally, it is important for the authors to justify their choice of countries, as despite being LMICs, these countries have unique characteristics that differentiate them from the vast majority of other LMICs.

- Authors' Response： We thank the reviewer for this comment. In response, we have now defined the acronym "NCD" (noncommunicable diseases) upon its first appearance in the title, abstract, and main text. We have also included the names of the analyzed countries (Benin,

Cameroon, Gabon, India, Madagascar, and Mauritania) in the abstract. Furthermore, all percentages throughout the manuscript have been standardized to one decimal place for consistency. The country selection criteria are detailed in the "Methods, data sources", and also addressed in our response to Reviewer 1 Comments 1 (" The authors have not explained the criteria or rationale of selecting these 6 countries out of all LMICs"). Specifically, these countries were chosen because they represent LMICs with complete and comparable information on both NCD indicators and SRH services. We believe these revisions have significantly improved the presentation of our work.

2 Contextualization and Discussion: The authors must discuss the impact of the COVID-19 pandemic on the indicators analyzed in Madagascar, including a review of studies that have already reported on this topic. Furthermore, the discussion lacks a detailed analysis of each of the estimated indicators (NCD prevention, contraceptive needs, antenatal care, and postnatal care) in the countries analyzed. A more granular discussion is needed, especially since numerous individual estimates of these topics already exist in the literature.

- Authors' Response： We thank the reviewer for this comment. In response, we have significantly strengthened the Discussion section. Specifically, in the country-specific analysis paragraph, we have incorporated a discussion on the impact of the COVID-19 pandemic in Madagascar. Given that the data for Madagascar were collected in 2021, we have cited relevant studies demonstrating how the pandemic adversely affected antenatal care utilization, providing crucial context for the high unmet need observed. Furthermore, in response to the reviewer's request for greater granularity, we have inserted two new country-level paragraphs into the Discussion.

Paragraph 4 (blood-pressure screening): we compare national performance and explain that Gabon and Cameroon achieve low unmet rates by integrating blood-pressure measurement into routine primary-care contacts, whereas Mauritania—plagued by shortages of basic equipment

and uneven staffing—records the highest never-measured proportion (53.8 %) among the six countries.

Paragraph 5 (blood-glucose testing): we show that overall unmet need is far higher (70.0 %) and dissect the cross-country gradient: Cameroon (65.5 %) and India (69.4 %) perform best, while Benin (91.9 %) and Madagascar (92.4 %) report that over nine in ten women have never been tested.

These additions sit in the newly created fourth and fifth Discussion paragraphs; the original country-specific analysis of cervical-cancer screening remains as paragraph 6. Consequently, every core indicator—contraception, ANC, PNC, blood-pressure, blood-glucose and cervical-cancer screening—now receives its own dedicated, country-by-country discussion, fully addressing the call for a more granular analysis.

3 Contextualization and Discussion: The authors must discuss the impact of the COVID-19 pandemic on the indicators analyzed in Madagascar, including a review of studies that have already reported on this topic. Furthermore, the discussion lacks a detailed analysis of each of the estimated indicators (NCD prevention, contraceptive needs, antenatal care, and postnatal care) in the countries analyzed. A more granular discussion is needed, especially since numerous individual estimates of these topics already exist in the literature.

- Authors' Response: We thank the reviewer for this comment. In response, we have significantly strengthened the Discussion section. Specifically, in the country-specific analysis paragraph, we have incorporated a discussion on the impact of the COVID-19 pandemic in Madagascar. Given that the data for Madagascar were collected in 2021, we have cited relevant studies demonstrating how the pandemic adversely affected antenatal care utilization, providing crucial context for the high unmet need observed. Furthermore, in response to the reviewer's request for greater granularity, we have inserted two new country-level paragraphs

into the Discussion.

Paragraph 4 (blood-pressure screening): we compare national performance and explain that Gabon and Cameroon achieve low unmet rates by integrating blood-pressure measurement into routine primary-care contacts, whereas Mauritania—plagued by shortages of basic equipment and uneven staffing—records the highest never-measured proportion (53.8 %) among the six countries.

Paragraph 5 (blood-glucose testing): we show that overall unmet need is far higher (70.0 %) and dissect the cross-country gradient: Cameroon (65.5 %) and India (69.4 %) perform best, while Benin (91.9 %) and Madagascar (92.4 %) report that over nine in ten women have never been tested.

These additions sit in the newly created fourth and fifth Discussion paragraphs; the original country-specific analysis of cervical-cancer screening remains as paragraph 6. Consequently, every core indicator—contraception, ANC, PNC, blood-pressure, blood-glucose and cervical-cancer screening—now receives its own dedicated, country-by-country discussion, fully addressing the call for a more granular analysis.

4 Data Source: Regarding the data source, it is puzzling that, given the recent events surrounding DHS funding, the authors do not comment—even in a note—on the impacts of the interruption of DHS research and the broader importance of this data source for studies on these topics in developing countries.

- Authors' Response： We thank the reviewer for raising this important point regarding the DHS data source. We fully acknowledge and share the concern about the critical importance of sustained DHS funding for public health research in developing countries. As such, we have explicitly expressed our gratitude to all country-level survey teams and participants in the Acknowledgements section of our manuscript.

The primary focus of our study, however, is to analyze the level and inequality of unmet needs for health services using the available DHS data. The specific impacts of funding interruptions on the DHS program itself, while a crucial topic for the global health community, fall outside the methodological and analytical scope of this particular paper. We agree that this is a vital issue worthy of dedicated discussion in other forums.

5 Unmet Contraceptive Needs: The unmet contraceptive needs indicator, although recommended by the WHO, is not the most widely used measure in the fields of public health and demography. I suggest that the authors justify their choice and also discuss the possibility of using other indicators, such as those discussed in the following texts. I believe that presenting the results of these studies and engaging in the methodological discussions would significantly strengthen the manuscript.

o BRADLEY, Sarah E.K.; CASTERLINE, John B. Understanding unmet need: history, theory, and measurement. Studies in family planning, v. 45, n. 2, p. 123-150, 2014.

o Bradley, Sarah E. K., Trevor Croft, Joy Fishel, and Charles Westoff. 2012. Revising Unmet Need for Family Planning. DHS Analytical Studies No. 25. Calverton, MD: ICF International.

o KARRA, Mahesh. Measurement of unmet need for contraception: a counterfactual approach. Studies in Family Planning, v. 53, n. 4, p. 657-680, 2022.

- Authors' Response: We thank the reviewer for this comment and have carefully viewed all the cited articles. We also cited relevant articles in our research to justify our choice. Considering the universal comparability and consistency with previous studies (Alattas et al., 2024, BMC Health Services Research), we employed the unmet need for contraception in our study. This approach ensures methodological coherence (Allin et al., 2010, Social Science & Medicine; Reeves et al., 2015, European Journal of Public Health; Bellizzi et al., 2020, Contraception; Adde et al., 2021, Reproductive Health; Yeboah et al., 2024, International Health) and allows for meaningful cross-country comparisons. We have explained our

definition in “Methods-Definitions” as follows: “Unmet contraceptive needs: Participants who reported “unmet need for spacing, unmet need for limiting” is classified as having unmet contraceptive needs, while the met need for contraception was defined as when a woman reported ‘using for spacing’ and ‘using for limiting’. This definition followed the previous consensus to measure the unmet needs.”

6 NCD Indicator Definition: Beyond this indicator, I believe the most complicated aspect is the definition used for the unmet needs indicator in NCD prevention, which includes blood pressure measurement, blood glucose measurement, and cervical cancer screening. I would like the authors to justify the relevance of these indicators, citing relevant studies on the topic. I believe that the issue of glucose and blood pressure testing is more specifically associated with pregnant women, not with all women in the study's age group. Furthermore, the chosen age group does not align with the recommended age for cervical cancer screening (women aged 25 to 64). I ask that the authors justify their decision to restrict the age group to women up to 49 years old. Why was breast cancer screening (recommended for women between 50 and 69 years old), a key preventive health test for women, not included? If a consensus on this indicator does not exist in the literature, it is essential for the authors to present the debate, justify their specific choices, and acknowledge the limitations that these choices entail. In other words, they need to acknowledge that the indicator fails to capture an important dimension of access to reproductive health.

- Authors’ Response: We thank the reviewer for this suggestion. The choice of indicators was primarily determined by the data availability in DHS datasets, which include questions on blood pressure measurement, blood glucose measurement, and cervical cancer screening. This operationalization is consistent with prior studies, such as Alattas et al. (2019), which defined unmet needs as reporting not seeing a physician in the past 12 months among those who were diagnosed with diabetes, hypertension, and dyslipidemia, and as not receiving cervical cancer

screening or a mammography. However, due to the absence of dyslipidemia and breast cancer screening indicators in the datasets of six selected countries, we only adopted three indicators, including blood pressure, blood glucose measurement, and cervical cancer screening.

The amended part in the "Methods-Definition" is as follows: "Unmet needs in NCDs prevention: Following Alattas's study, our definition of unmet needs in NCDs prevention is based on whether individuals have received the following screening services: blood pressure measuring, blood glucose measuring, or cervical cancer screening.

We acknowledge the reviewer's concern regarding the age coverage of cervical cancer screening. Although the WHO recommends cervical cancer screening for women aged 25–64, the DHS restricts individual-level data to women aged 15–49 years, which limited our analysis to this age group. Several previous DHS-based studies have adopted similar definitions within this age range (Refs. 21, 27, 28). The amended part in the "Methods-Definition" is as follows: "Besides, though the recommended age for cervical cancer screening is 25 to 64, the DHS only collected individual data for women aged 15 to 49. Consistent with other DHS-based studies, our study focused on the included groups."

We have clarified these limitations and added corresponding points in the Discussion section to acknowledge that our indicator may not fully capture all dimensions of NCD preventive service access as follows "Third, we acknowledge that the definition of unmet needs for breast cancer may not include women aged 25 to 64 recommended by the WHO due to the age restrictions in DHS data, thus weakening the representativeness".

7 Interpretation of Findings: Despite being seemingly low, the percentage of women with unmet contraceptive needs cannot be overlooked and must be discussed within the context of reproductive and sexual rights. The data on unmet antenatal and postnatal care services reflect a similar situation, given the extremely low fertility rates and persistently high maternal

mortality rates in these countries.

- **Authors' Response：** We thank the reviewer for this comment. We fully agree that quantitative prevalence should be interpreted in light of rights-based and public health contexts. In response, we have revised the second paragraph of the Discussion section to explicitly address the implications of the seemingly low unmet contraceptive needs. We now emphasize that a prevalence of 7.58% signifies a substantial denial of reproductive autonomy for a large number of women and poses serious health risks—particularly in settings characterized by low fertility and high maternal mortality. This addition firmly situates our findings within the framework of sexual and reproductive rights and aligns with the principles of the WHO's EMPOWER strategy. Furthermore, in the subsequent section discussing potential solutions, we have strengthened our recommendations to include a specific focus on enhancing women's empowerment and autonomy as a fundamental strategy to address these gaps.

8 Figures: Finally, the figures must be redone. They are currently illegible, even when magnified, which compromises the manuscript's presentation and clarity.

- **Authors' Response:** We thank the reviewer for this comment. The figures were prepared at the highest possible resolution; however, the reduced legibility may have resulted from file compression during the Word or PDF conversion process. We have now attached the original high-resolution figures for the editorial team's review and will be happy to make further adjustments according to the editors' technical specifications or recommendations.
